# BatteryBERT: A Pretrained Language Model for Battery Database Enhancement

**DOI:** 10.1021/acs.jcim.2c00035

**Published:** 2022-05-09

**Authors:** Shu Huang, Jacqueline M. Cole

**Affiliations:** † Cavendish Laboratory, Department of Physics, University of Cambridge, J.J. Thomson Avenue, Cambridge CB3 0HE, U.K.; ‡ ISIS Neutron and Muon Source, Rutherford Appleton Laboratory, Harwell Science and Innovation Campus, Didcot, Oxfordshire OX11 0QX, U.K.

## Abstract

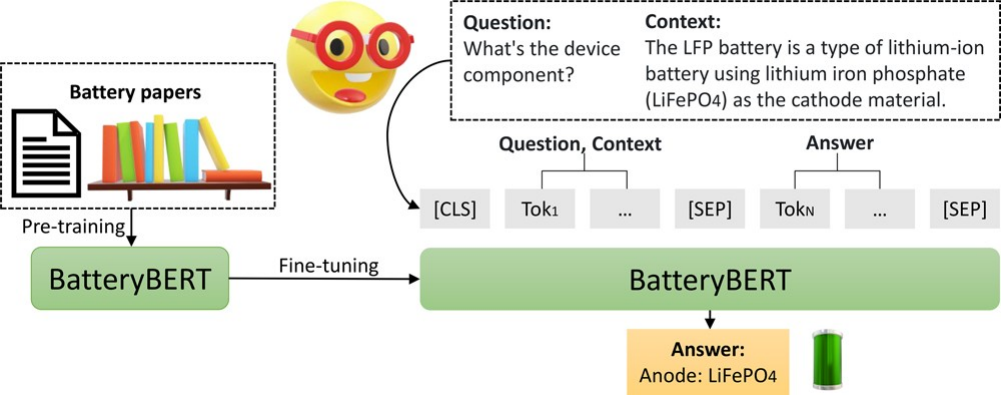

A great number of scientific papers are published every year in the field of battery research, which forms a huge textual data source. However, it is difficult to explore and retrieve useful information efficiently from these large unstructured sets of text. The Bidirectional Encoder Representations from Transformers (BERT) model, trained on a large data set in an unsupervised way, provides a route to process the scientific text automatically with minimal human effort. To this end, we realized six battery-related BERT models, namely, BatteryBERT, BatteryOnlyBERT, and BatterySciBERT, each of which consists of both cased and uncased models. They have been trained specifically on a corpus of battery research papers. The pretrained BatteryBERT models were then fine-tuned on downstream tasks, including battery paper classification and extractive question-answering for battery device component classification that distinguishes anode, cathode, and electrolyte materials. Our BatteryBERT models were found to outperform the original BERT models on the specific battery tasks. The fine-tuned BatteryBERT was then used to perform battery database enhancement. We also provide a website application for its interactive use and visualization.

## Introduction

The number of scientific publications in the area of battery research has been growing exponentially in recent years, but these huge textual resources are not being explored thoroughly by scientists. Yet, advancements in Artificial Intelligence and Natural Language Processing (NLP) are enabling NLP-based text-mining techniques to provide a way for rapid large-scale information retrieval and data extraction from the materials-science literature without too much human intervention.^1−6^ Language modeling is one of the NLP applications that has undergone considerable development in the past decade, and it lays the foundation of many other NLP tasks. The first-generation language models (Word2Vec,^7^ Glove,^8^ FastText^9^) have proven abilities in capturing semantic meanings of words. Meanwhile, the latest models are able to capture more complex concepts with minimal human labeling, especially the bidirectional long short-term memory (LSTM)-based models,^10^ and the transformer-based GPT-3^11^ and BERT.^12^

The latest developments of transformer-based models incorporate transfer learning into NLP, where a deep-learning model is trained on a large data set and the pretrained weights are fine-tuned for another task. This functionality enables transformers to be used in many downstream tasks, such as question answering or machine translation.^13^ Language models can also be domain specific. Once trained over a large corpus that is specific to a particular domain, such as scientific papers, language models can be used to perform tasks in that typical domain, for example, chemical-named entity recognition (CNER) in the chemistry area.^14−16^ There are many variants of domain-specific models, such as SciBERT,^17^ BioBERT,^18^ ClinicalBERT,^19^ and FinBERT.^20^ All of these models have been demonstrated to outperform the other BERT models on the downstream domain-specific tasks.

However, there is a lack of huge language models such as BERT in the field of scientific research. While BERT models have been used extensively in biomedical areas, they are rarely found in the domain of chemistry and materials science, especially in the field of battery research, in which millions of research papers have been published. A battery database was recently autogenerated from large-scale data extraction of scientific papers using the ”chemistry-aware” NLP tool, ChemDataExtractor.^21−23^ However, its construction was reliant on tedious manual labeling and training. We propose that BERT models are likely to improve the accuracy of battery data extraction and extend the functionalities of text mining in the area of battery research. One example of a functional extension is the extraction of relational data that can be extracted by fine-tuning the pretrained BERT model on a question-answering data set. If the training data set contains information about battery device components, the fine-tuned model can then be used for large-scale device-component classification and data extraction.

To this end, we trained and realized six battery-related BERT models for battery text mining, based on the battery papers from several publishers, including the Royal Society of Chemistry (RSC), Elsevier, and Springer. Our model is demonstrated to outperform the other BERT models on the battery text-mining tasks, such as question answering and battery paper classification. These fine-tuned models were then used to update and enhance a battery database that was created previously.^21^

## Method

**Figure 1:**
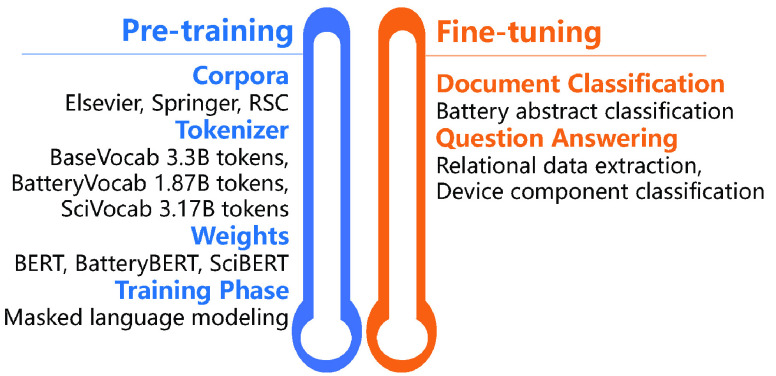
Two stages of the battery-related BERT models: pretraining and fine-tuning.

We developed six different battery-related BERT models for this work: BatteryBERT-cased, BatteryBERT-uncased, BatterySciBERT-cased, BatterySciBERT-uncased, BatteryOnlyBERT-cased, and BatteryOnlyBERT-uncased, based on different corpora that were used for pretraining. As is shown in Figure 1, we trained the BERT models on battery research papers either from previous weights or from scratch. Different vocabularies were used for different models. Once the pretraining stage had completed, the pretrained BatteryBERT models were fine-tuned for two downstream tasks: document classification, to distinguish the battery or nonbattery text, and question answering, to classify the battery device component. The details of each step will be introduced in this section, including model overview, text preprocessing, model pretraining and fine-tuning, and data extraction using the BatteryBERT models.

### Model Overview

The architecture of our battery-related BERT models (BatteryBERT, BatterySciBERT, and BatteryOnlyBERT) is consistent with that of the original BERT models for the ease of comparison.^12^ We also tried to keep the same parameters as the original BERT models, such as the number of hidden layers, number of attention heads, and vocabulary size. The key parameters used in our models are listed in Table 1. Both cased and uncased models were created for our battery-related BERT models. The cased model keeps the same text in the original papers as input, including both the capitalized and lowercase words, while the uncased models only use the words in lowercase. The main difference between the three types of models (BatteryBERT, BatterySciBERT, and BatteryOnlyBERT) is the training source. BatteryBERT was pretrained from the original BERT weights, in which the training data originate from English Wikipedia and Books Corpus,^24^ and then further trained in additional steps that employ our own data, that is, battery research papers.^21^

**Table 1:** Key Parameters of Battery-Related BERT Model

parameters	value
num_hidden_layers	12
hidden_size	768
num_attention_heads	12
attention_probs_dropout_prob	0.1
max_position_embeddings	512
total_number_of_parameters	∼110 million

**Figure 2:**
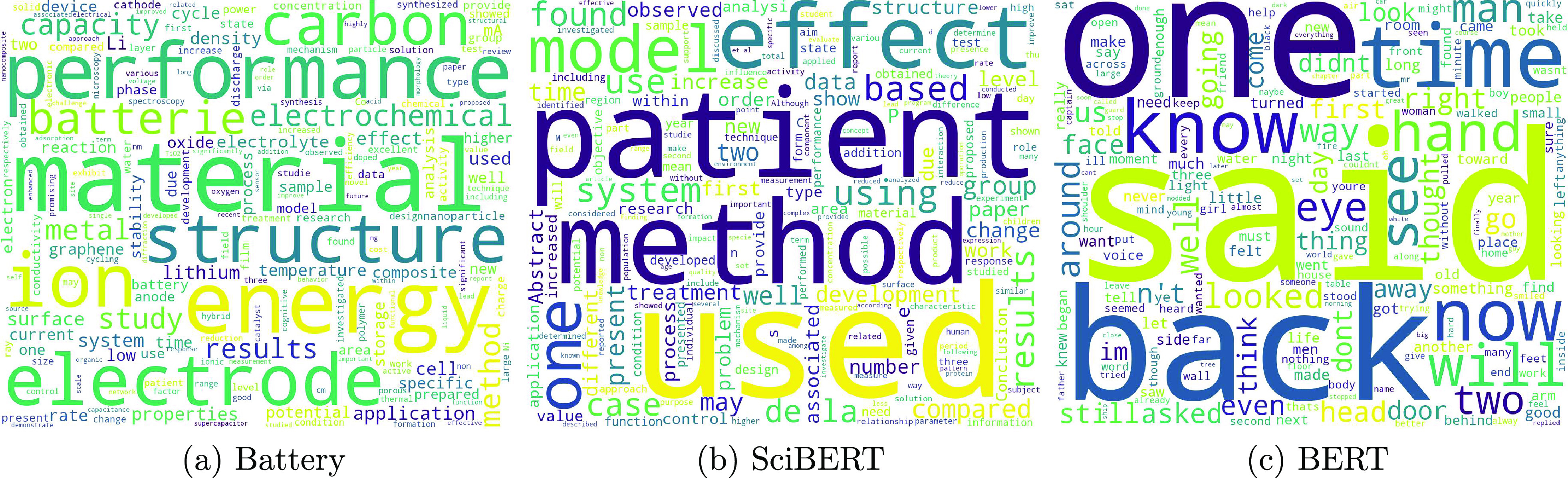
Word cloud of the most frequent words in the vocabularies of the (a) battery corpus, (b) SciBERT corpus, and (c) BERT corpus.

By contrast, the further training steps that afforded BatterySciBERT started from the SciBERT weights.^17^ SciBERT was mainly pretrained on biomedical and computer science-based scientific text, and SCIVOCAB was also used for tokenization rather than BASEVOCAB from the original BERT model. The last model, BatteryOnlyBERT, was trained from scratch using only our own paper-sourced data sets. The tokenizer of BatteryOnlyBERT was also trained exclusively from these battery research papers. Figure 2 shows the word cloud pictures of the three training corpora. It is clear that in our battery corpus, more battery-related words such as ”energy” and ”electrode” appear more frequently, whereas the other two corpora contain more biomedical-related or more general English words, such as ”patient” and ”said” for SciBERT and BERT, respectively.

### Text Preprocessing

**Figure 3:**
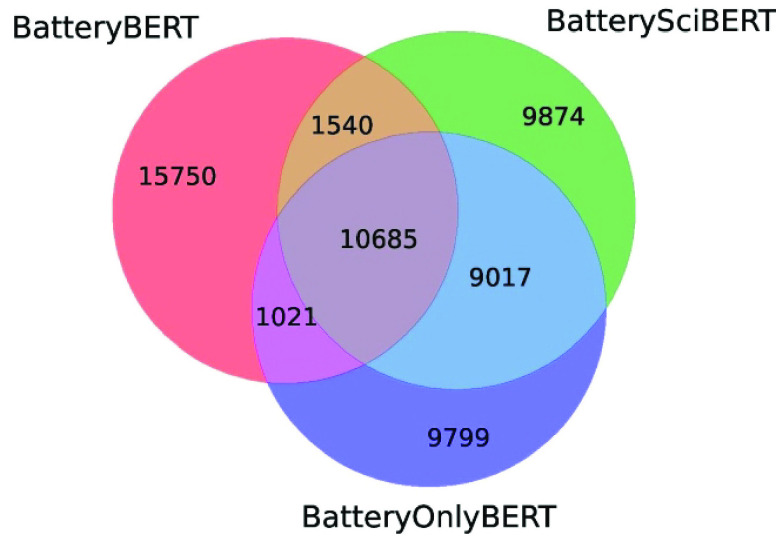
Comparison of the vocabularies of the BatteryBERT, Battery- SciBERT, and BatteryOnlyBERT models.

The battery research papers were retrieved using web-scraping tools from ChemDataExtractor^22,23^ and python HTTP client library ”requests”. More details of article retrieval have been provided by Huang and Cole.^21^ The literature data were initially downloaded in HTML/XML format and saved in text format, in order to be easily processed in the text-processing step. Then, a subword tokenizer, WordPiece tokenizer,^25^ was used to decompose rare words into smaller meaningful subwords. An example of a subword tokenizer is as follows: a single word ”*solvation*” is split into three words: *so*, *##l*, and *##vation*. In this way, the vocabulary size can be reduced to a reasonable level in order to improve the computational efficiency. The vocabulary files of BatteryBERT and BatterySciBERT are the same as the original BERT and SciBERT vocabulary, respectively; while the size of both vocabularies is the same as the original model. For BatteryOnlyBERT, we trained our own WordPiece tokenizer from the battery-paper data sets. Figure 3 shows the comparison of vocabularies of the three BERT models. The resulting BatteryVocab file of BatteryOnlyBERT shares 40.4% of the same words as the vocabulary file of the original BERT model, while around 60% of words in BatteryVocab are brand new and they are more about chemistry or batteries than the general popular English words that were used by the original BERT model. As shown in Figure 2a, the most common words in the word cloud picture of the battery corpus, such as *electrode*, *energy*, and *batteries*, demonstrate that the focus of this vocabulary file is in the domain of batteries. This also reflects that the text about battery research is a central part of the corpus that further trains the BatteryBERT and BatterySciBERT models.

**Figure 4:**
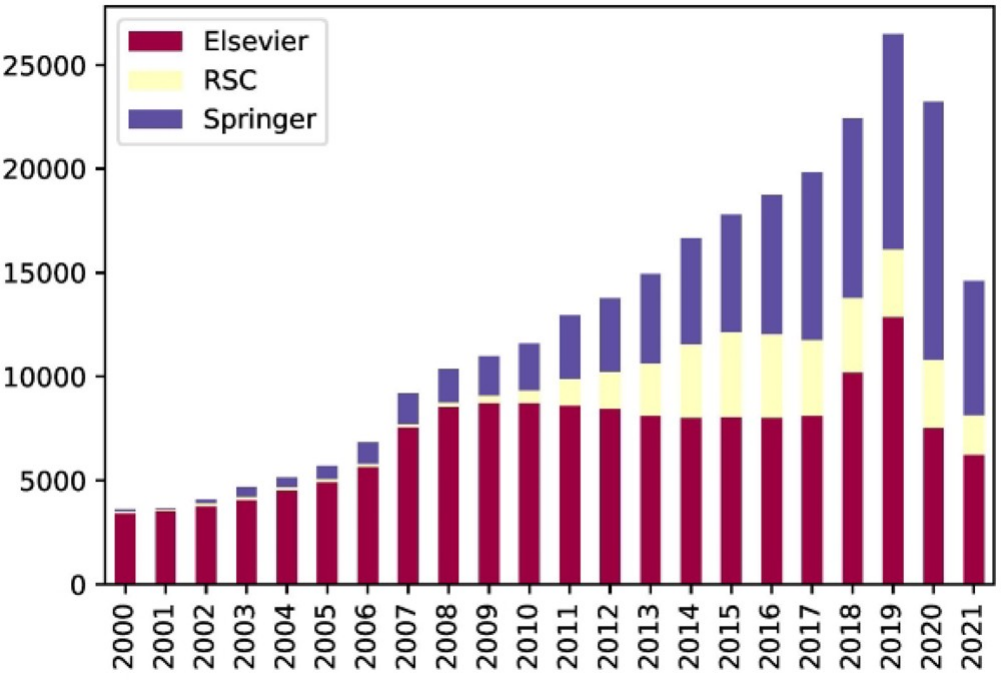
Stacked bar chart: the number of published papers about battery materials over the last 20 years. Entries for 2021 included data only up to June 2021 (inclusive) as the data in this study were extracted up to this time.

Overall, the number of individual word tokens from the corpus of battery papers from our previously created database^21^ is 1870 million, compared to 3300 million tokens that were used to train the original BERT model and 3170 million tokens that were employed to train SciBERT, as shown in Table 2. This also reflects the overall complexity of each model. Our training corpus includes scientific papers from three publishers: RSC, Elsevier, and Springer, whose total number over the years from 2000 to June 2021 is 400 366. Figure 4 shows the trend of papers about battery materials from different publishers over the last 20 years. Compared to our first autogenerated battery database,^21^ the number of RSC and Elsevier papers has been augmented by 12 905 and 74 213, respectively, over the last 2 years, while a brand new collection of 84 187 Springer papers have also been added, to enhance this original battery database.

**Table 2:** Number of Tokens Used to Train BatteryBERT, BatterySciBERT and BatteryOnlyBERT

model	number of tokens
BatteryBERT	3.3B + 1.87B
BatterySciBERT	3.17B + 1.87B
BatteryOnlyBERT	1.87B

### Pretraining and Fine-Tuning

Figure 1 shows two stages in which the three battery-related BERT models were trained: pretraining and fine-tuning. In the pretraining stage, the words of our corpus of battery papers were used for pretraining after initializing weights from either existing models or from scratch. The main training phase of the three models is masked language modeling (MLM), in which 15% of words in the employed corpus are masked and the transformer model is trained to predict the masked words. The next sentence prediction (NSP) loss is removed from the training objective, due to its unimproved performance, as suggested by Liu et al.^26^ We trained all of our battery-related BERT models with a batch size of 256, and the maximum sequence length was fixed to 512. The training time was 5 days for BatteryBERT and BatterySciBERT models (further trained for 1 000 000 steps) and 7 days for the BatteryOnlyBERT model (1 500 000 steps), using eight NVIDIA DGX A100 GPUs on the ThetaGPU cluster at the Argonne Leadership Computing Facility (ALCF). Details of the pretraining hyperparameters can be found in the Supporting Information.

Fine-tuning is a step to refine the large pretrained model which is then used to perform similar tasks on this or other data sets. This process is known as transfer learning, during which only minimal architectural changes are required for its use in downstream tasks within our operational pipeline. In other words, only the extra fully connected layers of the BERT model need to be trained and fine-tuned in this stage, while most of the parameters in the original model remain the same. As shown in Figure 1, the battery-related BERT models were used and examined on two downstream tasks: document classification and question answering. For these two tasks, most hyperparameters are the same as in pretraining, while the batch size, learning rate, and the number of the training epochs are changed. The optimal hyperparameters are task-specific, and we tested the following possible values for all tasks: batch size (16, 32), learning rate (Adam: 5 × 10^–5^, 3 × 10^–5^, 2 × 10^–5^), and the number of epochs (1–4 for Q&A, 1–15 for document classification). The details of these fine-tuning tasks will be discussed below.

#### Document Classification

BERT is able to perform document classification by adding a new sequence-classification layer on the top of the fine-tuning stage of the pretrained BERT model.^27^ In our study, document classification was performed to classify whether the research paper is relevant to battery research or not, according to the given abstract. In our original battery paper corpus, the papers were downloaded automatically using ChemDataExtractor.^22,23^ However, as the paper search in this scraping process was completed by just finding the word “battery” or “battery materials” that occurs in the original paper, the corpus of battery papers inevitably includes publications that are not about battery research. For example, a battery can be used in biomedical research, as written in a research paper (e.g., ref (28)), where the key of that research is not about battery materials. Therefore, it is necessary to perform document classification to filter out the irrelevant papers.

A training set is needed for fine-tuning the BatteryBERT models to classify battery and nonbattery papers. The training data set in this study was created by manually labeling journal names that belong to battery research or not. A labeled training data set was created from a total of 8137 journals that span different scientific areas. Journals whose names are in the area of battery research were labeled as “battery”, such as “Journal of Power Sources” or “Journal of Applied Electrochemistry”. By contrast, journals with names such as “Human Genetics” were labeled as “nonbattery”. A total of 1058 journal names were labeled manually, which contained a total of 46 663 papers in our corpus. Table 3 shows the number of battery papers (69.7%) and nonbattery papers (30.3%), respectively, in the data set that we created. It also shows the percentages of papers that were used in the training (70%), validation (20%), and test (10%) data sets. Note that the manual labeling of journals by their names can still include irrelevant papers; for example, solar cell research can also be mentioned in the electrochemistry journal. However, labeling journals by their names instead of individual papers that are published within them saves a lot of time without too much compromise when a large training-test data set is involved; such a data set is likely to be created in this way. To compare the performance of our models with that of traditional techniques, we also used the logistic regression (LR)-based binary-classification model as a baseline performance in our comparison.

**Table 3:** Training, Validation, and Test Datasets of the Document Classification

	battery papers	nonbattery papers	total
training	20 629	12 034	32 663
validation	5 895	3 438	9 333
test	2 948	1 719	4 667
total	29 472	17 191	46 663

#### Extractive Question Answering

**Figure 5:**
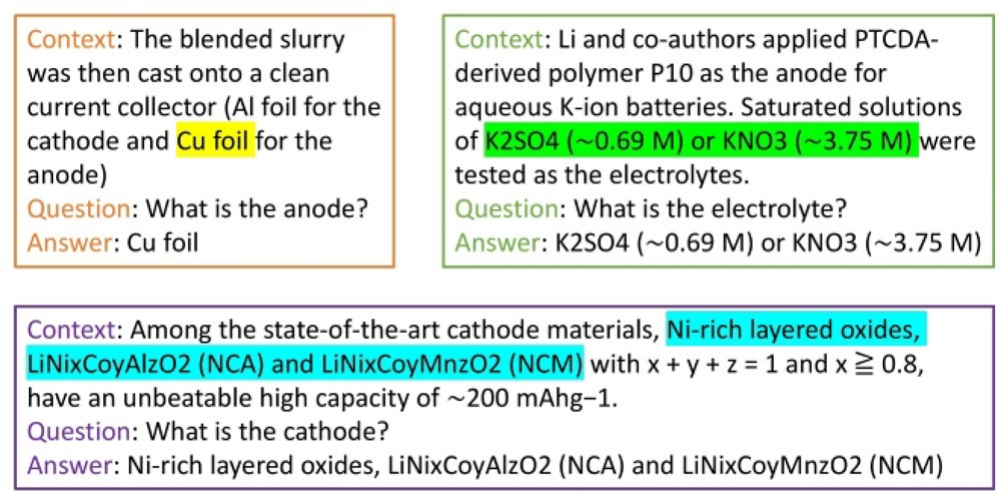
Examples of the evaluation question-answering data set.

The BERT models were also fine-tuned for an extractive question answering task in the battery domain. This task required a training data set of general question-and-answering (Q&A), for which the SQuAD v1.1 data set was used, together with our battery-related BERT models. SQuAD v1.1 includes >100 000 questions for machine comprehension in various domains.^29^ The newer SQuAD version 2.0^30^ was excluded in our fine-tuning objective, as the final use of the Q&A system is limited in the battery area and does not include unanswerable questions. We fine-tuned our battery-related BERT models from these >100 000 SQuAD records with a train/validation ratio of 90%:10%, and we also created and manually labeled a custom Q&A data set that is relevant to the battery domain for evaluation use. This specific Q&A data set has the same format as the SQuAD data set,^29^ and it contains three types of questions: “what is the anode?”, “what is the cathode?” and “what is the electrolyte?”. This data set consists of a total of 272 data records. Several examples of this evaluation data set are shown in Figure 5.

By adding a span-classification layer on the top of the fine-tuning stage of the battery-related BERT models, they can be fine-tuned to answer specific questions given the context. This kind of question-answering system is known as extractive question answering, since the answer is extracted from the words in their given context. Thus, the fine-tuned model can be used for relational data extraction. In addition, a confidence score can be calculated by the model, which indicates the confidence that the answer is the right match for the given question. This confidence score is given by the multiplication of the token level probabilities for the start and end location of answer phrases that are calculated from the softmax function. The equation is given by
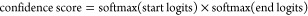
1where
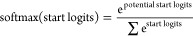
2and
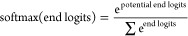
3In this way, the start and end tokens of the answers with the highest probability will be extracted. The confidence score can then be used as a threshold in the data-extraction step to adjust the precision/recall trade-off. To deal with the specific Q&A cases for battery materials, the chemical-named entity recognition (CNER) capabilities of ChemDataExtractor were used to filter the answers that are not a valid chemical name. Similarly, a name-normalization data-cleaning step was also implemented to remove invalid material property data.

### Data Extraction

**Figure 6:**
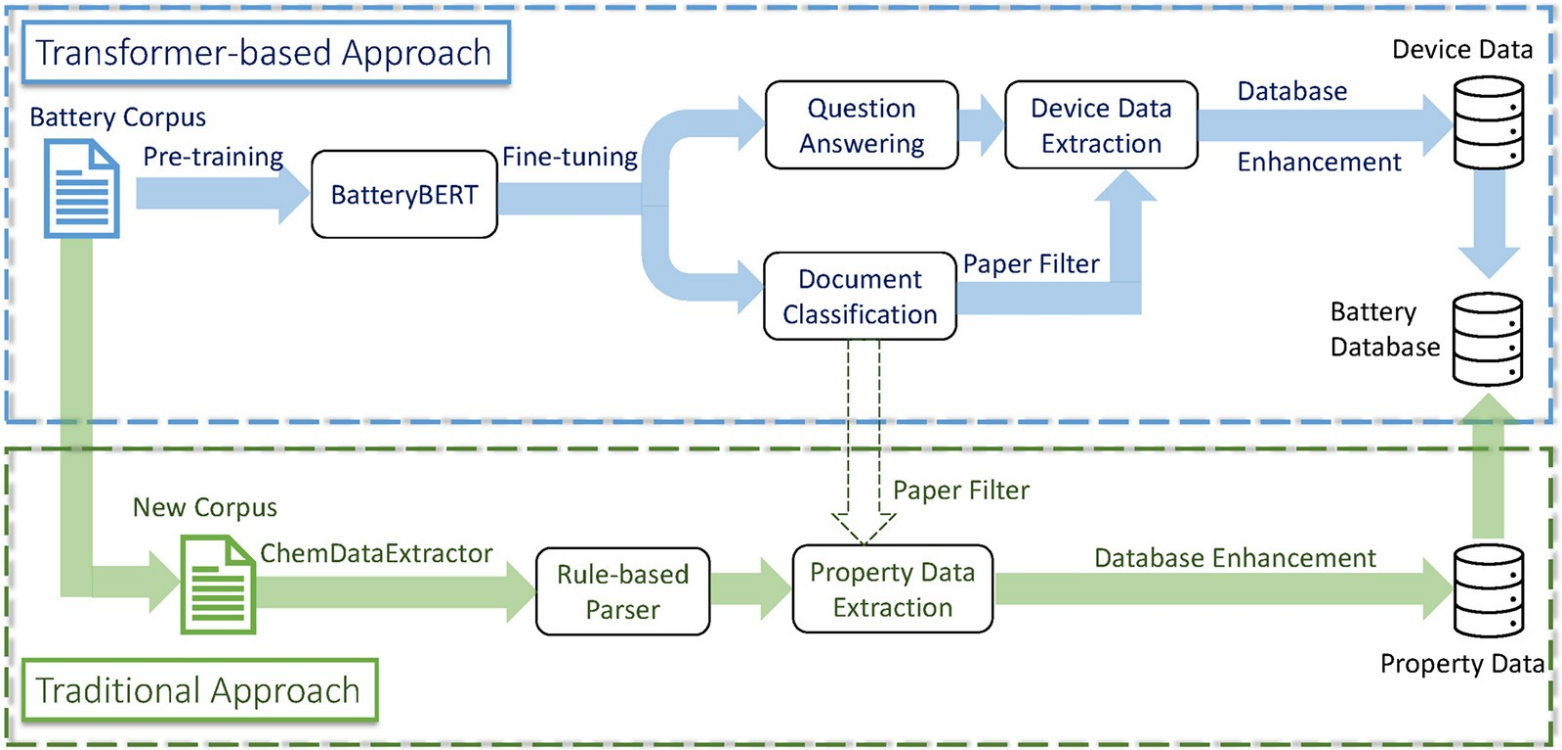
Database enhancement pipeline: transformer-based approach and traditional approach.

Figure 6 shows the database enhancement pipeline in this research, including both the transformer-based and traditional approach. In the transformer-based approach, the preprocessed text data from the set of battery papers were first pretrained from scratch or from BERT weights to create a BatteryBERT model. The BatteryBERT model was then fine-tuned on two downstream tasks, document classification and question answering, as introduced above. The document classifier was used as a nonrelevant paper filter, so that the nonbattery papers could be removed from the corpus of papers. The fine-tuned BatteryBERT contained an additional Q&A layer at its front end in order to perform the database enhancement step as a preprogrammed Q&A agent. In this step, the words of the battery device components (anode, cathode, electrolyte) were detected automatically by searching for the exact words in the paper. Once the words “anode”, “cathode” or “electrolyte” were detected, the preprogrammed questions about these device materials were asked, and the battery device components data were then classified using the fine-tuned preprogrammed model. The fine-tuned preprogrammed model includes a default confidence score threshold, which was determined by testing the different performance of each confidence-score parameter from 0 to 1 on our manually labeled battery-specific data set in terms of the precision-recall trade-off. The extracted device data were finally saved into the battery database, thereby resolving each material therein with its device function.

For the traditional approach, we reapplied the battery property parser which was previously applied to extract the data that formed the originally autogenerated battery-materials database,^21^ this time to a new corpus of literature that was either published more recently than the data extraction of the original battery-material database, or was sourced from a previously unsorted publisher. The document classifier that has been described in the transformer-based approach was also used in this step to remove nonrelevant data. The new collection of \{material, property\} data were then combined with the device-classified data and eventually saved into the battery database in the final step.

## Results

### Model Performance

#### Pretraining

We pretrained our models for 1 000 000 steps for BatteryBERT and BatterySciBERT, and 1 500 000 steps for BatteryOnlyBERT since this model was trained from scratch. The number of training steps was determined according to the original BERT model, which was also trained for 1 M steps,^12^ and the authors claimed that BERT was trained for 1 M steps, and it achieved better performance for language modeling tasks when trained on 1 M steps compared to 500 K steps. The number of epochs is around 46 for BatteryBERT and BatterySciBERT models, while the training epochs for the BatteryOnlyBERT model is ∼70.

**Figure 7:**
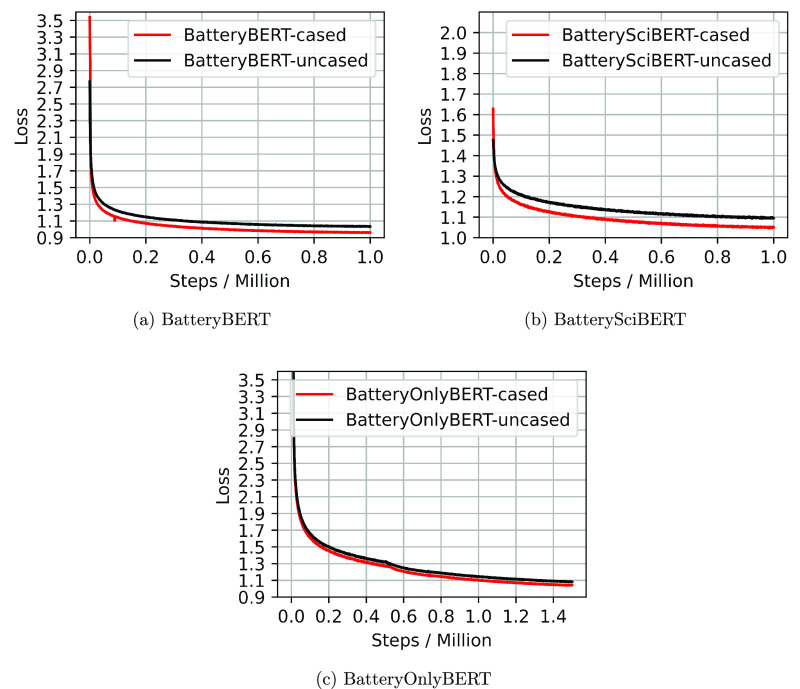
Loss versus pretraining steps for the BatteryBERT, BatterySciBERT, and BatteryOnlyBERT models.

Figure 7 shows how the loss varies with the training steps. The final loss for all models lies in the range between 0.96 and 1.09, and the loss values of all the uncased models is slightly higher than those of the cased models. Since the BatteryOnlyBERT model was trained only on the battery text from scratch, the pretraining loss converges slower than that in the other models. Overall, the training loss keeps decreasing during these training steps, and the models that stop pretraining at this point are demonstrated to have good performances on the downstream tasks as will be introduced later.

#### Document Classification

Table 4 presents the accuracy of binary document classification of the test data set for each model. The logistic regression (LR) classifier used term frequency-inverse document frequency (Tf-idf) features as input and achieved an accuracy of 90.95% and 90.12%, for cased and uncased model, respectively; this is regarded as the baseline performance for accuracy metrics of the binary classification for the various BERT models, which are shown in Table 4. Binary classification for all BERT models outperforms the traditional approach (LR) by ∼6–7%, which proves that by using the deep-learning based pretrained language models, one can achieve much better results than when using the traditional machine learning techniques.

**Table 4:** Accuracy of Binary Classifiers for the Test Dataset of Battery and Nonbattery Abstracts

model	LR	BatteryBERT	BatterySciBERT	BatteryOnlyBERT	Original BERT
accuracy cased	90.95%	96.85%	97.19%	97.34%	96.83%
accuracy (uncased)	90.12%	96.94%	**97.47%**	97.08%	96.29%

Binary classification shows almost the same performance between BERT models within a very narrow range of accuracy scores. Still, all of the battery-related BERT models outperform the original BERT models, demonstrating the positive effect of domain-specific pretraining on downstream tasks. The BatterySciBERT-uncased model achieved the highest accuracy for this task, which might be due to greater overlap with the SciBERT vocabulary. This model will be used as the final model for the battery paper classification.

#### Extractive Question Answering

To examine the performance of the extractive question-answering system, we used two evaluation metrics: exact match-single (EM-single) and F1-single score, on the SQuAD data set. These scores are computed on individual question-and-answer pairs. The EM-single score is the percentage of the extracted data with exactly the same answers as those in the evaluation data set. Therefore, EM-single can only be either 1 or 0 for an individual record. The F1-single score is calculated via the precision-single and recall-single metrics, as given by
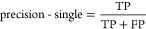
4
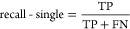
5
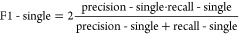
6where TP denominates true positive, FP is false positive, and FN is false negative.

As the answer of a question often contains multiple words, a true positive metric signifies the percentage of words that is shared between the original true answer and the predicted answer; false positive is defined as the percentage of words that are in the predicted answer but not in the true answer; and false negative is the percentage of words that are in the true answer but not in the predicted answer. Note that the EM-single and F1-single score are only determined from a single answer, and a final value of EM-single and F1-single score are obtained by averaging over the individual scores. Therefore, we name the metrics as EM-single and F1-single. The EM-single and F1-single score can be used to evaluate the prediction performance of the model. Compared to the EM-single score, the F1-single score reflects the model performance more accurately. For example, one of the predicted answers for the electrolyte materials can be “KOH and 0.2 M ZnAc_2_”, while the true answer in the training set can be “A mixed solution of 6 M KOH and 0.2 M ZnAc_2_”. In this case, the EM-single score is calculated as 0 since these two answers are not exactly the same, but the F1-single score is 0.615 as the predicted answers contain some of the same words in the true answer (TP, 4/9; FP, 0/9; FN, 5/9; precision-single, 1; recall-single, 4/9; F1-single, 0.615).

Table 5 reports the performance of our battery-related BERT models on the SQuAD validation data set in comparison with the original BERT model. Both the cased and uncased BatteryBERT achieved the highest EM-single score and F1-single score compared to the other cased/uncased model. This demonstrates that the further pretraining of the original BERT on the battery-specific domain can also improve the performance on the general English Q&A data set. However, the performance of BatterySciBERT and BatteryOnlyBERT models is worse than the original BERT model, which is as expected as these models were not pretrained on the general English corpus such as books and Wikipedia.

**Table 5:** EM-Single and F1-Single Score of Each Model for the SQuAD Validation Dataset

model	EM-single score	F1-single score
BERT-base-cased	81.30	88.58
BatteryBERT-cased	**81.54**	**89.16**
BatterySciBERT-cased	79.66	87.43
BatteryOnlyBERT-cased	79.61	87.30
BERT-base-uncased	80.93	88.20
BatteryBERT-uncased	**81.08**	**88.41**
BatterySciBERT-uncased	79.81	87.66
BatteryOnlyBERT-uncased	79.53	87.22

To demonstrate the utility of our battery-related BERT models in the specific domain of battery materials, we evaluated all models on the manually labeled Q&A data set, including 272 battery device data. After the question about battery device materials was answered, we used the NLP function of ChemDataExtractor_batteries^21^ to filter out the invalid material data, and a confidence score threshold to filter out the data that is less likely to be true. We used two evaluation metrics for this battery-specific evaluation data set: precision-data set and recall-data set, both of which are calculated based on the whole evaluation data set. Recall-data set is the proportion of the material data that has been answered and extracted from the 272 evaluation data records with a higher confidence score than the threshold, while precision-data set is the average value of the F1-single scores of each individual extracted answer. Recall-data set can reflect how versatile the model is to answer questions under different contexts, while precision-data set can be used to evaluate the prediction accuracy of the model.

Using the evaluation metrics above, the performance of the different battery-related BERT models on our manually labeled data set can be compared. As shown in Table 6, the BatteryBERT model has the highest precision-data set score for both cased (70.74%) and uncased (68.27%) models among all of the models, and both models also have a relatively high recall-data set score (84.17% and 80.88%, respectively). For the cased model, the original BERT model shows a slightly higher precision-data set score compared to the BatterySciBERT and BatteryOnlyBERT models, but it has the lowest recall-data set score. By contrast, for the uncased model, all of the three battery-related models have higher precision-data set and recall-data set score compared to the original uncased BERT model, while the BatterySciBERT-uncased model achieved the highest recall-data set score (85.29%) among the eight models. Like the document classification task, this finding also demonstrates that further pretraining from the BERT and SciBERT weights or pretraining only on battery text from scratch can help to achieve better results in the domain-specific task. Note that for all these BERT models, most of the wrong answers were extracted from very long contextual text, since the BERT model is better at dealing with short textual extracts (e.g., less than 512 words). The misclassification of CNER for complicated material names also led to worse model performance. Thus, our battery-related BERT models can exhibit even better performance on shorter textual data sets when using better CNER tools.

**Table 6:** Precision-Dataset and Recall-Dataset Score of Each Model on the Manually Labeled Battery-Specific Dataset

model	precision-data set	recall-data set
BERT-base-cased	67.02	80.15
BatteryBERT-cased	**70.74**	84.19
BatterySciBERT-cased	65.09	**84.56**
BatteryOnlyBERT-cased	64.28	82.72
BERT-base-uncased	62.19	75.00
BatteryBERT-uncased	**68.27**	80.88
BatterySciBERT-uncased	66.65	**85.29**
BatteryOnlyBERT-uncased	67.20	83.82

**Figure 8:**
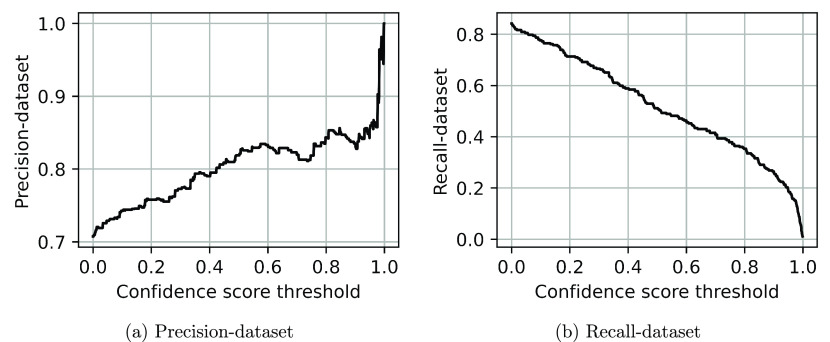
Precision-data set and recall-data set versus the confidence score threshold for the BatteryBERT-cased model.

As the BatteryBERT-cased model has the highest precision-data set score, and third highest of recall-data set score, it was chosen to be used for the device component classification and large-scale data extraction. To determine the best parameters, we evaluated the performance of the BatteryBERT-cased model as a function of the confidence score threshold on our manually labeled battery-specific data set. Each confidence score was calculated, as shown in eq 1. Figure 8 shows how the confidence score threshold varies with the precision-data set and recall-data set score; when the confidence score threshold is larger than 0.4, the precision-data set score is 79.01%. At this point, the recall-data set score (58.82%) is also relatively high. Therefore, a confidence score threshold of 0.4 for the BatteryBERT-cased model was set as the final model for the further database enhancement.

### Visualization

**Figure 9:**
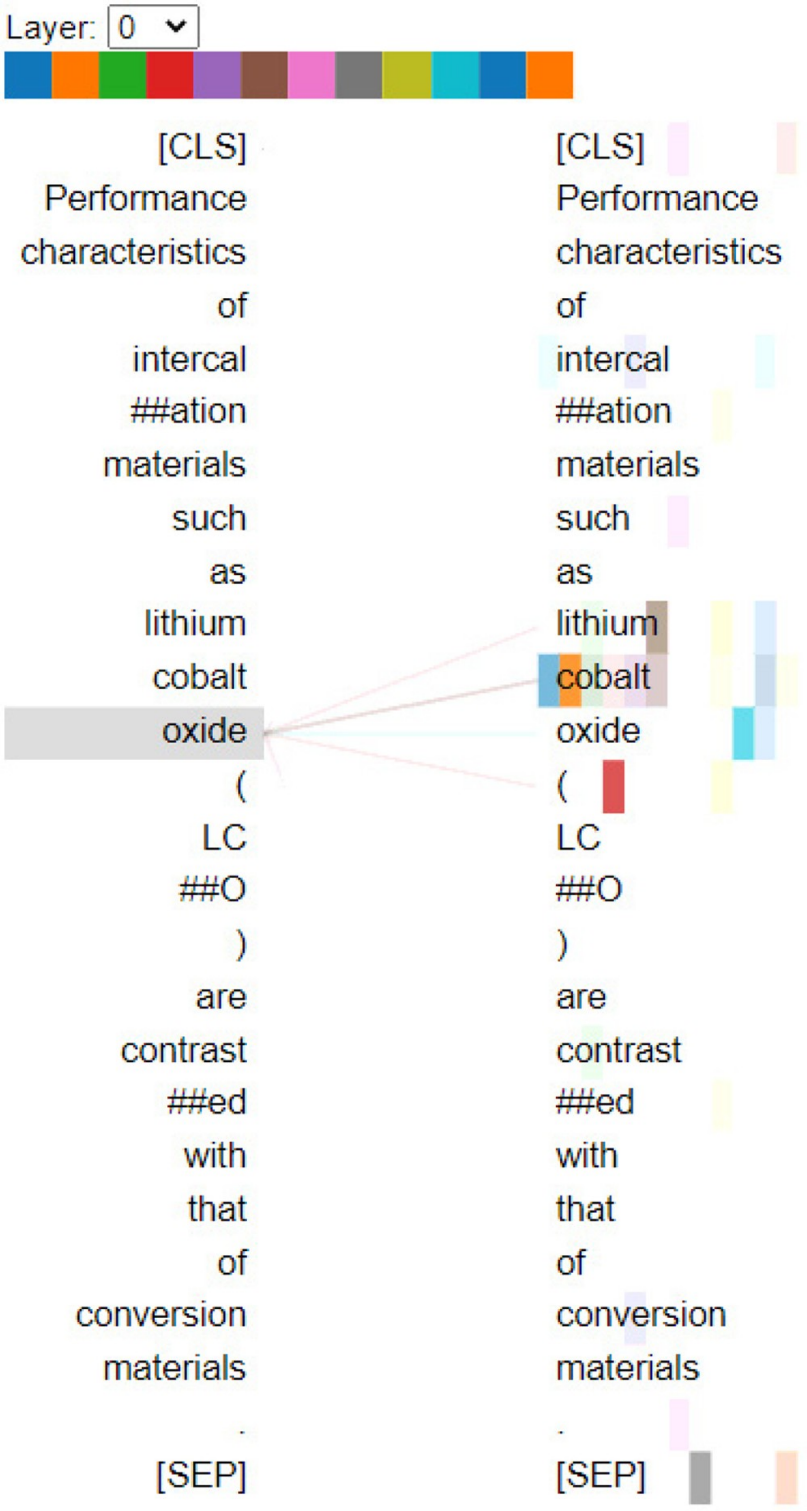
Visualization of Layer 0 in the BatterySciBERT-cased model architecture. (https://huggingface.co/batterydata/batteryscibert-cased).

An intuitive view of what attention patterns the battery-related BERT models have learned was obtained using the bertviz^31^ tool. Transformer-based models such as BERT use a multilayer, multihead architecture in which these attention patterns are generated. Bertviz enables one to visualize these patterns by breaking down this complicated part of the BERT architecture into a series of multiple-layer bipartite graphs for a given sentence of text. Thereby, each token of a sentence represents a node (vertex) on each side of the graph; while the edges that join them represent the nature and strength of attention patterns that link each token to another within the sentence. Each edge carries a weighting that quantifies the extent of attention (the attention score) between two tokens that results from the combination of the many attention patterns that describe how each pair of tokens is semantically related to other words in the sentence by its context. The contextual information from each sentence is embedded into a BERT model via a set of attention heads, the number of which is fixed for a given model, as defined by the user input. This study employed 12 Heads, each of which is identifiable by color assignment (cf. the color bar at the top of Figure 9). Each Head performs a linear transformation of each token once the token has been vectorized into its own token embedding. The linear combination of the token and contextual embedding produces an updated token vector, the values of which differ from the original vectorized token in a numerical fashion that captures the influence of the sentence content on this token. Figure 9 illustrates a typical result, whereby the token “oxide” is highlighted in gray in the sentence given on the left of Figure 9; the same sentence is given on the right of this bipartite graph, although the coloring of tokens in this sentence is multivariate; each color thereupon represents the color of a Head which has a significant influence (contextual bearing) on the token “oxide”. Thus, Figure 9 reveals that “oxide” is highly correlated to cobalt and, to a lesser extent, lithium, as one would expect for a sentence about the compound, lithium cobalt oxide, in the BatteryBERT model; i.e., “oxide” is strongly related to the chemical constituents of the compound to which it belongs and this compound is highly contextually related to battery materials. Moreover, “oxide” is correlated to [SEP] which separates a sentence, or sentence fragment, from another that is otherwise classified [CLS].

**Figure 10:**
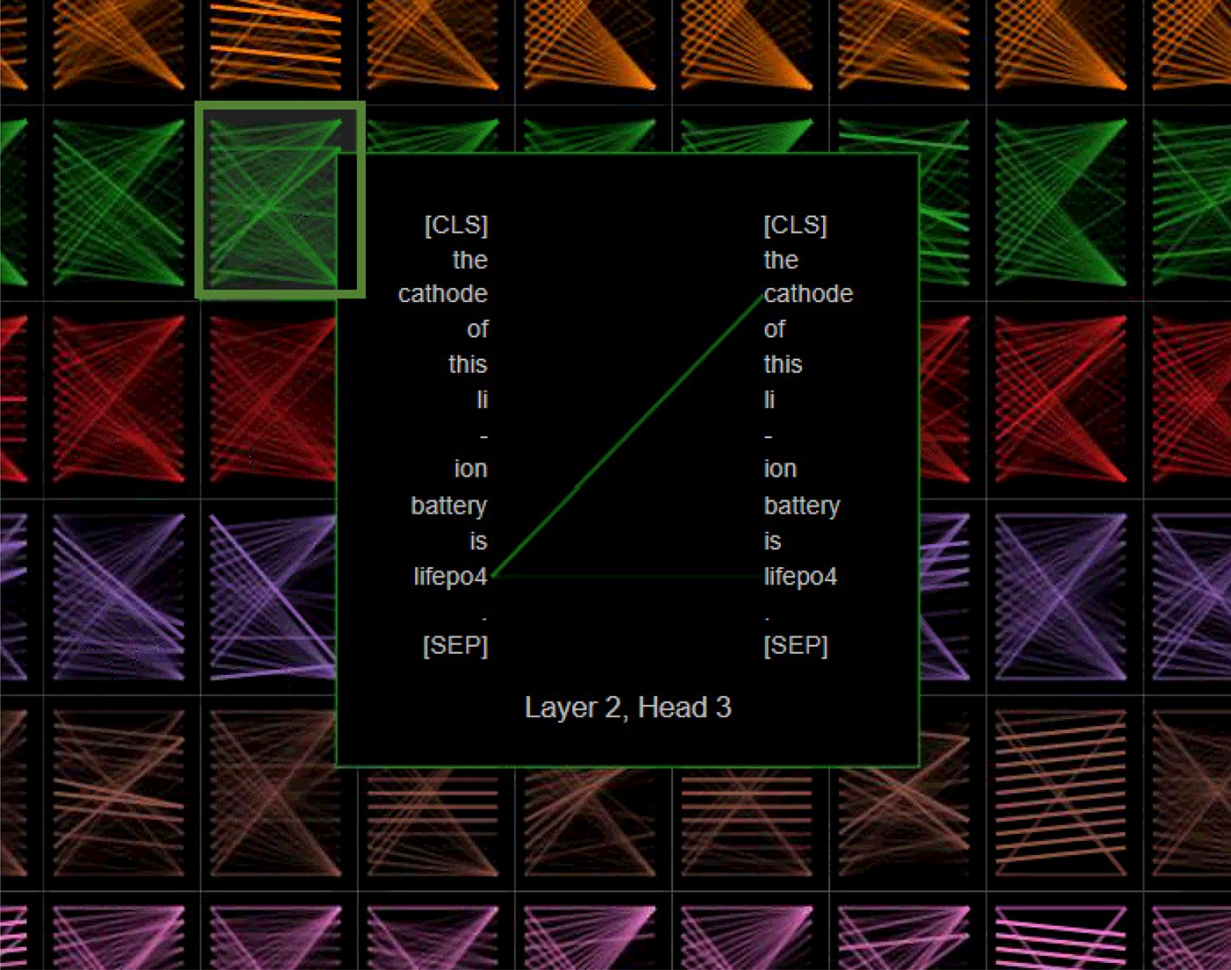
Visualization of Layer 2, Head 3 of the fine-tuned Q&A BatteryOnlyBERT-uncased model architecture. (https://huggingface.co/batterydata/batteryonlybert-uncased-squad-v1).

Aside from providing a token-level perspective of attention patterns within a BERT model, the Bertviz tool also provides a view of the attention patterns that span the full multilayer, multihead model via its display thumbnail images of attention patterns within an *m* × *n* array of *m* Layers (rows) × *n* Heads (columns). The image for each Layer (row) represents the *n*th time that a fuller version of the aforementioned token embedding modification procedure in the BERT architecture is repeated, in order to afford a final model. Figure 10 shows this model view for attention patterns of the Q&A fine-tuned BatteryOnlyBERT-uncased model when it employed 12 Layers and 12 attention Heads. Therein, its (Layer 2, Head 3) attention patterns are illustrated using the example sentence “The cathode of this Li-ion battery is LiFePO4”. If this sentence is passed for contextual information for the extractive Q&A task, “lifepo4” points to the word “cathode”. Given the battery-specific nature of such patterns that form in our models, we believe that this visualization tool helps to evidence that BatteryBERT models can capture the contextual features after their pretraining and fine-tuning steps.

### Battery Corpus and Database Updates

The fine-tuned BatteryBERT model was first used as a binary classifier to partition battery and nonbattery related papers that were downloaded from the publishers. Table 7 lists the results for the paper classification. Around 62.5% of the total number of papers were removed from our corpus of papers, most of which are not in the domain of battery research but still mentioned the word “battery” in the paper. However, some battery papers can be misclassified as “nonbattery” papers when the paper does not have an abstract, or the metadata is too old to parse by ChemDataExtractor. More than 82.5% of the RSC papers remained as battery-related papers following this filtering process, which might be due to the greater chemistry-specific focus of RSC publications; while the other two publishers are much more multidisciplinary and contain many papers from a wider range of scientific domains, such that the filtering process removed many more of them (see Table 7).

**Table 7:** Classification of Battery or Nonbattery Papers from the Three Publishers Using the Fine-Tuned BatteryBERT Model

	RSC	Elsevier	Springer	total
battery papers	36 794	91 513	21 685	149 992
nonbattery papers	7 800	180 072	62 502	250 374
total	44 594	271 585	84 187	400 366

Having performed property data extraction on the new collection of battery-material containing papers (published in RSC or Elsevier journals from 2019 until June 2021 or published in Springer journals), these data were then added to the battery-material database. The resulting augmented database was compared with the originally created battery-material database.^21^Table 8 presents the changes to the original database owing to the battery-relevance filters and the data extracted from new battery text sources. While the number of conductivity and Coulombic efficiency data increases by a small amount when the paper filter and Springer data are included, owing to the most recently published papers added, the other three property data diminish in quantity. The large reduction in the total number of data is caused by the voltage data, which alone accounts for 54.8% of the total reduction. The rationale for this appears to be that voltage can be mentioned in various applications, such as a biomedical device, while the other four properties, especially conductivity and Coulombic efficiency, are most likely to be used to only describe battery materials, bearing in mind the paper search criteria that had been employed. Therefore, once the nonbattery papers had been removed by our binary-classification filters, there is a huge reduction in the voltage data, but the other property data are not affected that much. Table 8 also lists a summary of the breakdown of property data in the new database, including a differentiation that signifies the contribution of the Springer data. A total of 231 345 property data were extracted from the papers that were published up to June 2021.

**Table 8:** Comparison between the Database with and without Paper Filter from RSC and Elsevier, and the Database with Paper Filter and with Springer Data

property	no. of data records (without paper filters, without Springer data)	no. of data records (with paper filters, with Springer data)	no. of data records (with paper filters, without Springer data)
capacity	144 359	139 118	129 232
conductivity	7 168	12 697	11 992
coulombic efficiency	11 033	12 692	11 821
energy	15 543	14 771	13 542
voltage	115 240	52 067	46 197
total	292 313	231 345	212 784

**Figure 11:**
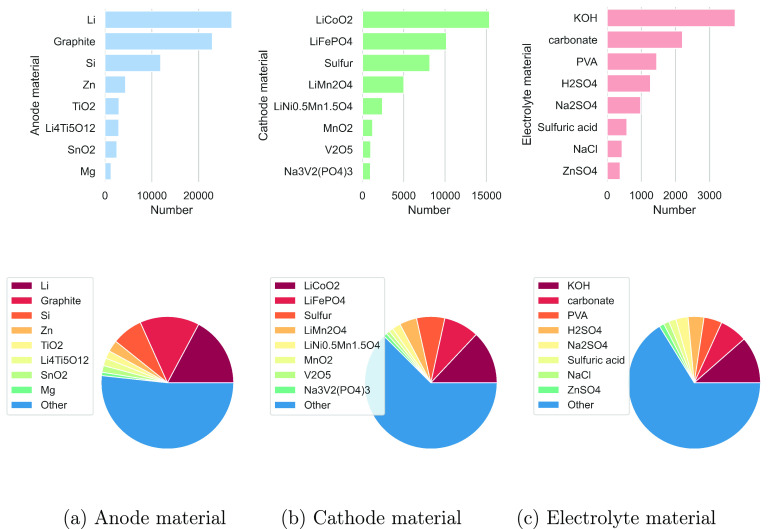
Distribution of device component materials and its top eight representatives.

A battery device component database was also created, consisting of 308 964 valid data including the classification of anode, cathode, and electrolyte materials. Table 9 shows a summary of data records in this device component database, while Table 10 lists the number of duplicate and unique data records of anode, cathode, and electrolyte materials. The large proportion of duplicates indicates that many of the same device component materials are used by different scientists. Among the 14 052 unique data records, a total of 11 960 unique materials were found, as some materials can be any of the three types of device components. For example, graphite is found to be used as either an anode^32^ or cathode^33^ in different types of batteries. Figure 11 gives an overview of the chemical distributions of anodes, cathodes, and electrolytes in the device material database. It also specifies the distribution of the top eight representative materials of each type of battery device component. Therefore, it is easy to find the most popular anode (Li), cathode (LiCoO_2_), and electrolyte (KOH) materials that were studied over recent years.

**Table 9:** Summary of Data Records in the Device Component Database

data	description	data type
type	device component types	string
name	chemical compound names	string
extracted_name	normalized chemical name	list of dictionaries
score	confidence score	float
context	context of original data	string
DOI	source article DOI	string
journal	published journal	string
date	published date	string
title	source article title	string

**Table 10:** Number of Data Records and Unique Data Records for Anode, Cathode, and Electrolyte Materials

types	no. of device records	no. of unique device records
anode	157 885	6 210
cathode	118 310	6 179
electrolyte	32 769	1 663
total	308 964	14 052

### Online DEMO

**Figure 12:**
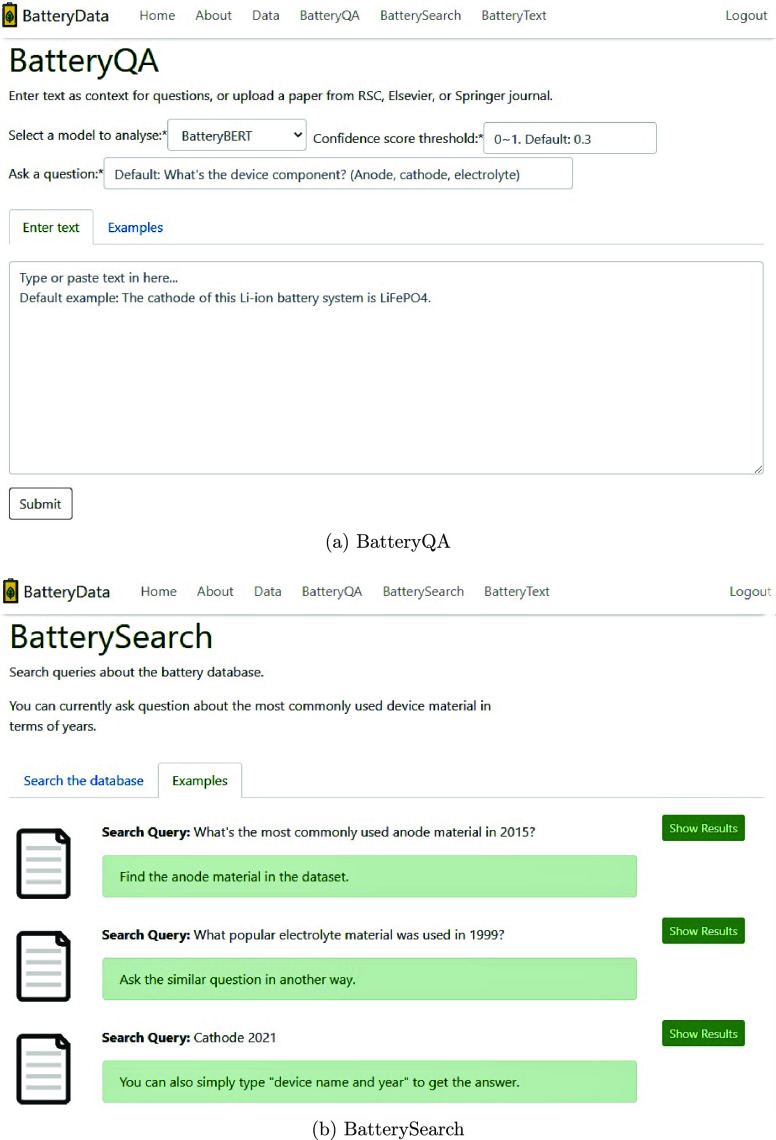
Snapshot of the BatteryData web app with BatteryQA and BatterySearch sections. (http://www.materialsforbatteries.org/).

To demonstrate the potential utility of BatteryBERT models, we created a Web site application (web app) that can be accessed via www.materialsforbatteries.org. Figure 12 presents the snapshot of two of its sections that are related to the battery Q&A system. The BatteryQA section has been designed for interactive use of the extractive Q&A fine-tuned BatteryBERT models. Users can select parameters on their own, including which one of the three BatteryBERT model options they wish to analyze, the confidence score threshold, the question to be asked (the default is a device component question), and the context of either a paper file or raw text. This web app will return the relevant device component materials if it finds an answer. The BatterySearch page is a section just for simple demonstration use just now. Accordingly, this Q&A system is based on the preprogrammed data set rather than the context that is provided each time. At present, questions such as “What is the most common electrolyte in 2019?” can already be answered since the relational data \{year, device component, material\} is saved in the back-end data sets. We hope to extend the functionality of this search-engine system by improving the preprogrammed data sets in the future. In addition, the web app also presents a database overview and an introduction about the relevant techniques that underpin this online tool. Overall, we hope that it can aid users to reuse, review, and visualize our battery database and models.

## Conclusion

To summarize, we have pretrained and released six battery-related BERT models: BatteryBERT, BatteryOnlyBERT, and BatterySciBERT, including both cased and uncased models. All of these models were pretrained on our battery-paper corpora; the main difference between the models is the initialized weights. Our battery-related BERT models outperform the original BERT models on two downstream tasks: document classification and extractive question answering. The BatteryBERT-cased model performs the best on the extractive Q&A task, while the BatterySciBERT-uncased model achieved highest accuracy for the document classification task. This indicates that further pretraining on a domain-specific area can improve the model performance compared to the original BERT model. The fine-tuned BatteryBERT-cased model was optimized on the Q&A data set for the purpose of device component classification to serve as a functional application. The visualized attention mechanism also indicates that there are battery-specific interpretable patterns behind the model weightings. We further employed the fine-tuned BatteryBERT-cased model to perform data extraction in order to enhance the originally released battery database.^21^ To the best of our knowledge, this is the first transformer-based language model that has been trained on the specific area of battery research, and it is also one of the first models that has been applied in the domain of scientific papers which are beyond the biomedical area.^34,35^ All of our pretrained and fine-tuned models have been deposited on https://huggingface.co/batterydata which can be easily accessed by the transformer library.^36^

Through this study, we have demonstrated that pretraining in an unsupervised way is able to achieve state-of-the-art results in text mining within a specific field without too much human intervention. Also, with minimal changes in model architecture during the fine-tuning step, BatteryBERT models can be used in many downstream battery-specific tasks. Owing to the nature of transfer learning, the limited amount of time required during fine-tuning increases the possibility of the BatteryBERT model to be applied to various research areas, even though the overarching BERT-based model is large and complicated. We believe that scientists can use our models to gain a thorough view of current battery research, and are also able to perform many battery-specific text-mining tasks except for just document classification and extractive question answering.

One limitation of this type of research is that performance can still be improved. For example, the overall performance of any task in this study could be improved by employing the large-BERT models, which feature more layers and more attention heads. Due to its larger architecture and more parameters, large-BERT is likely to achieve better results even when using the same training set. We only selected the base-BERT architecture for this study, since the use of large-BERT models would have cost so many computational resources that it would discourage the use of our models owing to their long running times. In addition, our models were pretrained from 400 366 papers; this number can be larger when more papers are published in the future, or when more publishers provide us the access to their papers. With more papers being trained on our models, BatteryBERT is likely to become a more professional “battery expert”. Also, our battery-related BERT models were found to be error-prone when dealing with longer contextual text, due to the nature of the model’s architecture, but it is still better than when using traditional methods, including the LSTM model. In addition, our battery-related BERT models were only fine-tuned on two specific tasks for this study, while many other tasks, such as chemical-named entity recognition (CNER), can also be tested using our models. Lastly, it is still a challenge for our battery-related BERT models to obtain implicit information using purely BERT models, which is in common with the other current language models that have proven abilities to capture the contextual information and comprehend the language text.

Overall, our battery-related BERT models have outperformed the original BERT model and achieved state-of-the-art results on the specific battery data set. Its usefulness is also demonstrated in text mining within the battery area; as an example, a more informative battery database can be created using the BatteryBERT-cased model. The battery-related BERT models will be further trained in the future, once more new papers have been published. The models and battery database created by the BatteryBERT-cased model are easily accessible via our web app for interactive use as well as its fine-tuning use on other particular tasks.

## Data and Software Availability

The pretraining and fine-tuning code was written using PyTorch and Transformers library^36^ and is available at the GitHub repository https://github.com/ShuHuang/batterybert, which also includes the code of BatteryBERT usage and data extraction. A list of DOIs for the papers that were used to train our models can be found in this GitHub repository to enable the reproducibility of this work. The code of ChemDataExtractor_batteries (v1.5) that has been modified for database autogeneration in the battery domain is available at https://github.com/ShuHuang/batterydatabase. All the BatteryBERT models and data sets used for fine-tuning can be found at https://huggingface.co/batterydata. The new battery property database v2.0 and device-classified battery database have been saved in JSON, SQL, and CSV format; both can be found on Figshare at 10.6084/m9.figshare.18154715.

## Supporting Information Available

Implementation details; BatteryBERT model usage; evaluation details of the paper abstracts data set; question-answering data set; evaluation details of the SQuAD data set; document classification data (PDF)
