# Supplementary material for: BatteryBERT: A Pretrained Language Model for Battery Database Enhancement
Source: J Chem Inf Model. 2022 May 9;62(24):6365–77. doi: 10.1021/acs.jcim.2c00035 (PMC9795558; doi:10.1021/acs.jcim.2c00035)
Supplement: Supplementary file 1 — ci2c00035_si_001.pdf [file ci2c00035_si_001.pdf]

Supporting Information: BatteryBERT: A Pretrained Language Model for Battery Database Enhancement

Shu Huang† and Jacqueline M. Cole*,†,‡

†*Cavendish Laboratory, Department of Physics, University of Cambridge, J.J. Thomson Avenue, Cambridge CB3 0HE, U.K.*

‡*ISIS Neutron and Muon Source, Rutherford Appleton Laboratory, Harwell Science and Innovation Campus, Didcot, Oxfordshire, OX11 0QX, UK.*

E-mail: jmc61@cam.ac.uk

S1. Implementation Details

1.1 Language Model Pretraining

The language model pretraining followed the masked language modeling (MLM) objective and used the BertForPreTraining architecture (a multi-layer bidirectional transformer encoder) from transformers. The model sizes used were: L=12, H=768, A=12, Total parameters = 110M, where L is the number of layers, H is the hidden size, A is the number of self-attention heads. 15% of the tokens had been masked at random. We trained with a batch size 256 and a maximum sequence length of 512. We used Adam with a learning rate of 2e-5, for the further pretraining of BatteryBERT and BatterySciBERT; and a learning rate of 1e-4, for the pretraining of BatteryOnlyBert from scratch. BatteryBERT and BatterySciBERT were further pretrained for 1,000,000 steps, while BatteryOnlyBert was pretrained for 1,500,000 steps. The training time was 5 days for BatteryBERT and BatterySciBERT (further trained for 1,000,000 steps) and 7 days for BatteryOnlyBert (1,500,000 steps), using eight NVIDIA DGX A100 GPUs on the ThetaGPU cluster at the Argonne Leadership Computing Facility (ALCF).

The other configuration and training parameters were:

- attention_probs_dropout_prob: 0.1
- hidden_act: gelu
- hidden_dropout_prob: 0.1
- initializer_range: 0.02
- intermediate_size: 3072
- layer_norm_eps: 1e-12
- max_position_embeddings: 512

- position_embedding_type: absolute
- transformers_version: 4.17.0.dev0
- no_cuda: False
- n_gpu: 8
- adam_beta1=0.9
- adam_beta2=0.999
- adam_epsilon=1e-08
- L2_weight_decay=0.01
- vocab_size: 28996 (cased), 30522(uncased)

1.2 Fine-tuning: Document Classification

In the fine-tuning stage, most model hyperparameters used were the same as in pretraining. The main differences were as follows. We used the "BertForSequenceClassification" as the model architecture. We used a dropout probability of 0.1 and optimize cross entropy using Adam. We fine-tuned 15 epochs using a batch size of 16 and 32, a learning rate of 2e-5, 3e-5 and 5e-5. The fine-tuning of document classification took around 1 hours on a single NVIDIA DGX A100 GPU.

1.3 Fine-tuning: Question Answering

Most of the model hyper-parameters used were the same as those employed in the pretraining and fine-tuning of document classification. We used the "BertForQuestionAnswering" as the model architecture in this step. We used a dropout probability of 0.1 and fine-tuned 4 epochs using a batch size of 16 and 32, a learning rate of 2e-5, 3e-5 and 5e-5. The fine-tuning of question answering took around 15 minutes on a single NVIDIA DGX A100 GPU.

1.4 Data Extraction

The document classifier and question-answering agent were combined with the traditional property data-extraction algorithm to create the battery database. For a subset of 10,000 papers, we used 10 nodes (CPUs) of ALCF Cooley systems with 3 sub-processes and it took 12 hours for the data extraction.

S2. BatteryBERT Model Usage

It is easy to use the BatteryBERT models using the transformers library from Hugging Face. Examples are given below.

Example usage of the language model:

```
from transformers import AutoTokenizer, AutoModelForMaskedLM
model_name = "batterydata/batterybert-cased"
tokenizer = AutoTokenizer.from_pretrained(model_name)
model = AutoModelForMaskedLM.from_pretrained(model_name)
```

Example usage of the document classification model:

```
from transformers import AutoTokenizer, AutoModelForSequenceClassification
class_model_name = "batterydata/batteryscibert-uncased-abstract"
tokenizer = AutoTokenizer.from_pretrained(class_model_name)
model = AutoModelForSequenceClassification.from_pretrained(
    class_model_name)
```

Example usage of the Q&A model:

```
from transformers import AutoTokenizer, AutoModelForQuestionAnswering
qa_model_name = "batterydata/batterybert-cased-squad-v1"
tokenizer = AutoTokenizer.from_pretrained(qa_model_name)
model = AutoModelForQuestionAnswering.from_pretrained(qa_model_name)
```

All of the models can be found at https://huggingface.co/batterydata/.

S3. Evaluation Details of the Paper Abstracts Data Set

	**Accuracy (Batch Size 16; Learning Rate 2e-5)**	**Accuracy (Batch Size 16; Learning Rate 3e-5)**
Bert-base-uncased	96.61 Epoch 11	96.79 Epoch 11
BatteryBERT-uncased	96.95 Epoch 12	96.61 Epoch 8
BatterySciBERT-uncased	96.85 Epoch 6	96.19 Epoch 13
BatteryOnlyBert-uncased	97.04 Epoch 14	97.18 Epoch 13
Bert-base-cased	96.62 Epoch 10	96.74 Epoch 13
BatteryBERT-cased	96.77 Epoch 10	96.45 Epoch 12
BatterySciBERT-cased	96.83 Epoch 14	96.30 Epoch 12
BatteryOnlyBert-cased	97.12 Epoch 11	96.89 Epoch 6
	**Accuracy (Batch Size 32; Learning Rate 2e-5)**	**Accuracy (Batch Size 32; Learning Rate 3e-5)**
Bert-base-uncased	96.79 Epoch 13	96.70 Epoch 13
BatteryBERT-uncased	97.10 Epoch 11	96.96 Epoch 7
BatterySciBERT-uncased	97.12 Epoch 14	97.00 Epoch 13
BatteryOnlyBert-uncased	97.17 Epoch 11	97.12 Epoch 6
Bert-base-cased	96.84 Epoch 15	96.53 Epoch 15
BatteryBERT-cased	97.29 Epoch 11	96.98 Epoch 9
BatterySciBERT-cased	97.06 Epoch 11	96.73 Epoch 13
BatteryOnlyBert-cased	97.03 Epoch 14	97.19 Epoch 11
	**Accuracy (Batch Size 16; Learning Rate 5e-5)**	**Accuracy (Batch Size 32; Learning Rate 5e-5)**
Bert-base-uncased	95.57 Epoch 13	96.54 Epoch 11
BatteryBERT-uncased	95.65 Epoch 2	96.47 Epoch 2
BatterySciBERT-uncased	95.00 Epoch 1	96.76 Epoch 13
BatteryOnlyBert-uncased	96.39 Epoch 1	96.67 Epoch 2
Bert-base-cased	95.48 Epoch 15	96.58 Epoch 11
BatteryBERT-cased	95.76 Epoch 1	96.25 Epoch 15
BatterySciBERT-cased	95.35 Epoch 1	95.53 Epoch 2
BatteryOnlyBert-cased	96.16 Epoch 1	96.91 Epoch 12

S4. Evaluation Details of the SQuAD Data Set

		**Batch Size 16; Learning Rate 2e-5**			
		**Epoch 1**	**Epoch 2**	**Epoch 3**	**Epoch 4**
Bert-base-uncased	EM	78.77	80.1	80.4	80.11
	F1	86.72	87.88	87.9	87.67
BatteryBert-uncased	EM	78.98	80.12	80.58	80.23
	F1	86.95	88	88.09	88.02
BatterySciBert-uncased	EM	78.64	79.51	79.44	79.41
	F1	86.71	87.38	87.36	87.35
BatteryOnlyBert-uncased	EM	77.87	79.53	79.24	78.88
	F1	86.11	87.22	87.22	87.19
Bert-base-cased	EM	79.37	80.94	80.78	80.81
	F1	87.17	88.52	88.37	88.45
BatteryBert-cased	EM	79.96	81.84	81.54	81.54
	F1	87.68	89.05	88.99	89.16
BatterySciBert-cased	EM	77.7	79.8	79.2	79.47
	F1	86.11	87.27	87.15	87.44
BatteryOnlyBert-cased	EM	77.44	79.43	79.61	79.4
	F1	85.28	86.56	87.3	86.98
		**Batch Size 16; Learning Rate 3e-5**			
		**Epoch 1**	**Epoch 2**	**Epoch 3**	**Epoch 4**
Bert-base-uncased	EM	79.21	80.05	80.6	80.18
	F1	87.23	87.93	88.18	87.98
BatteryBert-uncased	EM	78.96	80.1	80.64	79.94
	F1	87.13	88.1	88.13	87.87
BatterySciBert-uncased	EM	78.07	78.92	79.78	79.28
	F1	86.35	87.01	87.28	87.31
BatteryOnlyBert-uncased	EM	77.75	79.06	79.34	78.6
	F1	85.9	86.95	87.22	86.99
Bert-base-cased	EM	79.67	80.61	80.86	80.4
	F1	87.28	88.17	88.39	88.31
BatteryBert-cased	EM	79.71	81.42	81.2	81.45
	F1	87.38	88.75	88.52	88.91
BatterySciBert-cased	EM	77.33	79.26	79	79.25
	F1	85.96	86.92	87.13	87.11
BatteryOnlyBert-cased	EM	75.87	79.81	79.53	79.21
	F1	84.36	87.01	86.97	86.91

		Batch Size 16; Learning Rate 5e-5			
		**Epoch 1**	**Epoch 2**	**Epoch 3**	**Epoch 4**
Bert-base-uncased	EM	78.82	79.43	80.05	79.04
	F1	86.54	87.55	87.96	87.34
BatteryBert-uncased	EM	78.12	78.93	80.02	79.8
	F1	86.16	87.29	87.64	87.76
BatterySciBert-uncased	EM	77.2	77.96	78.87	78.43
	F1	85.49	86.34	86.79	86.79
BatteryOnlyBert-uncased	EM	76.98	77.91	78.15	77.79
	F1	85.22	85.96	86.39	86.57
Bert-base-cased	EM	79.42	80.14	80.76	80.36
	F1	86.89	87.78	88.33	88.34
BatteryBert-cased	EM	78.66	80.47	80.67	80.74
	F1	86.44	87.73	88.31	88.39
BatterySciBert-cased	EM	76.56	78.48	78.52	78.53
	F1	85.04	86.63	86.61	86.94
BatteryOnlyBert-cased	EM	76.58	78.82	78.47	78.45
	F1	84.64	86.11	86.18	86.51
		**Batch Size 32; Learning Rate 2e-5**			
		**Epoch 1**	**Epoch 2**	**Epoch 3**	**Epoch 4**
Bert-base-uncased	EM	78.01	79.24	80.06	79.76
	F1	86.35	87.26	87.61	87.62
BatteryBert-uncased	EM	78.86	79.75	80.76	80.48
	F1	86.75	87.92	88.17	88.12
BatterySciBert-uncased	EM	77.6	79.47	79.81	79.64
	F1	86.11	87.27	87.66	87.51
BatteryOnlyBert-uncased	EM	76.87	79.07	79.18	78.94
	F1	85.35	86.81	87.05	86.96
Bert-base-cased	EM	78.99	80.68	80.86	80.58
	F1	86.71	88.2	88.24	88.25
BatteryBert-cased	EM	79.85	81.05	81.17	81.25
	F1	87.66	88.5	88.71	88.81
BatterySciBert-cased	EM	76.79	79.17	79.66	79.56
	F1	85.5	86.77	87.43	87.24
BatteryOnlyBert-cased	EM	77.2	79.28	79.45	79.21
	F1	85.36	86.65	87	86.86

		Batch Size 32; Learning Rate 3e-5			
		**Epoch 1**	**Epoch 2**	**Epoch 3**	**Epoch 4**
Bert-base-uncased	EM	79.1	79.78	80.93	79.94
	F1	86.91	87.67	88.2	87.75
BatteryBert-uncased	EM	79.36	80.13	81.08	80.39
	F1	87.05	88.17	88.41	88.22
BatterySciBert-uncased	EM	78.08	79.47	79.74	79.46
	F1	86.59	87.28	87.53	87.48
BatteryOnlyBert-uncased	EM	77.53	79.14	79.18	78.7
	F1	85.68	86.79	87.18	86.97
Bert-base-cased	EM	79.5	81.14	80.78	80.5
	F1	87.2	88.51	88.3	88.31
BatteryBert-cased	EM	79.37	80.89	81.36	81.04
	F1	87.06	88.41	88.66	88.65
BatterySciBert-cased	EM	76.88	79.51	79.21	79.26
	F1	85.65	87.14	87.34	87.3
BatteryOnlyBert-cased	EM	76.99	79.74	79.56	79.23
	F1	85.3	86.99	87.27	87.14
		**Batch Size 32; Learning Rate 5e-5**			
		**Epoch 1**	**Epoch 2**	**Epoch 3**	**Epoch 4**
Bert-base-uncased	EM	78.63	79.95	80.45	79.23
	F1	86.73	87.89	87.96	87.61
BatteryBert-uncased	EM	79.33	79.62	80.4	80.26
	F1	87.02	87.58	87.99	88.16
BatterySciBert-uncased	EM	77.04	78.99	78.98	79.03
	F1	85.42	86.72	87.02	87.09
BatteryOnlyBert-uncased	EM	76.83	79.63	79.15	78.31
	F1	85.08	87.07	87.23	86.72
Bert-base-cased	EM	79.73	81.3	80.58	80.18
	F1	87.34	88.58	88.31	88.12
BatteryBert-cased	EM	79.59	80.88	81.11	80.8
	F1	87.23	88.2	88.54	88.53
BatterySciBert-cased	EM	76.88	78.68	78.59	79.09
	F1	85.41	86.53	86.71	87.12
BatteryOnlyBert-cased	EM	75.45	77.86	78.67	78.46
	F1	83.81	85.67	86.35	86.32

S5. Question Answering Data Set

The full data set can be found at

`https://huggingface.co/datasets/batterydata/battery-device-data-qa`

Examples of the question answering evaluation data set are given below:

	Question	Answer	Context	Start index
1	What is the cathode?	Al foil	The blended slurry was then cast onto a clean current collector (Al foil for the cathode and Cu foil for the anode) and dried at 90 °C under vacuum overnight.	645
2	What is the anode?	Cu foil	The blended slurry was then cast onto a clean current collector (Al foil for the cathode and Cu foil for the anode) and dried at 90 °C under vacuum overnight. Finally, the obtained electrodes were cut into desired shapes on demand. It should be noted that the electrode mass ratio of cathode/anode is set to about 4, thus achiev -ing the battery balance.	673
3	What is the cathode?	SiC/RGO nanocomposite	In conclusion, the SiC/RGO nanocomposite, integrating the synergistic effect of SiC flakes and RGO, was synthesized by an in situ gas–solid fabrication method. Taking advantage of the enhanced photogenerated charge separation, large CO2 adsorption, and numerous exposed active sites, SiC/RGO nanocomposite served as the cathode material for the photo-assisted Li–CO2 battery.	284
...	...	...	...	...

S6. Document Classification Data Set

The document classification data set includes 29,472 battery papers and 17,191 non-battery papers. These papers are manually labeled in terms of the journals to which they belong. 14 battery journals and 1,044 non battery journals were selected to form this database.

Battery journals:

Applied Energy	Battery Reference Book
Electrochimica Acta	Energy & Environmental Science
Journal of Applied Electrochemistry	Journal of Electroanalytical Chemistry
Journal of Energy Chemistry	Journal of Energy Storage
Journal of Power Sources	Nano Energy
Nature Energy	Renewable Energy
Renewable and Sustainable Energy Reviews	
Renewable Energy Conversion, Transmission and Storage	

Non-battery journals:

3-D Fibrous Assemblies
A Baker's Dozen
ADHD Attention Deficit and Hyperactivity Disorders
AIDS Research and Therapy
Accident Analysis & Prevention
Acoustics Australia
Acta Analytica
Acta Geodaetica et Geophysica
Acta Mathematica Scientia
Acta Mechanica Solida Sinica
Acta Metallurgica Sinica (English Letters)
Acta Neuropathologica
Acta Otorrinolaringológica Española
Acta Tropica
Acta Veterinaria Scandinavica
Actas Dermo-Sifiliográficas (English Edition)
Advanced Modeling and Simulation in Engineering Sciences
Advanced Textiles for Wound Care
Advances in Applied Mechanics
Advances in Ceramic Matrix Composites

Advances in Climate Change Research
Advances in Developmental Biology and Biochemistry
Advances in Food Traceability Techniques and Technologies
Advances in Health Sciences Education
Advances in Insect Physiology
Advances in Non-Volatile Memory and Storage Technology
Advances in Parasitology
Advances in Smart Medical Textiles
Advances in Sugarcane Biorefinery
Agricultural and Forest Meteorology
Air & Space Europe
Allergo Journal International
Alzheimer's & Dementia: Translational Research & Clinical Interventions
American Journal of Ophthalmology
Amino Acids
An Introduction to Biochemistry
Analog Synthesizers
Animal Biotelemetry
Annales de Chimie Science des Matériaux
Annales de Chirurgie Vasculaire
Annales de Génétique
Annales de Réadaptation et de Médecine Physique
Annals of Clinical Microbiology and Antimicrobials
Annals of Surgical Oncology
Annals of Vascular Surgery
Annual Reports Section "B" (Organic Chemistry)
Anthropocene
Applied Biochemistry and Biotechnology
Applied Clay Science
Applied Coal Petrology
Applied Computing and Informatics
Applied Dimensional Analysis and Modeling
Applied Geochemistry
Applied Mathematics & Optimization
Applied Mycology and Biotechnology
Applied Process Design for Chemical & Petrochemical Plants
Applied Soft Computing

Applied and Preventive Psychology
Applying Knowledge Management
Aquaculture International
Architecture of Network Systems
Archive for Rational Mechanics and Analysis
Archives de Pédiatrie
Archives of Dermatological Research
Archives of Osteoporosis
Archives of Pharmacal Research
Archives of Physical Medicine and Rehabilitation
Argumentation
Assessment and Treatment of Child Psychopathology and Developmental Disabilities
Astrophysics and Space Science
Atención Familiar
Atención Primaria
Atmospheric Environment
Audio Wiring Guide
Australasian Marketing Journal (AMJ)
Automated Lighting
Automation and Remote Control
Autonomous Agents and Multi-Agent Systems
Autophagy: Cancer, Other Pathologies, Inflammation, Immunity, Infection, and Aging
Avery's Diseases of the Newborn
BMC Blood Disorders
BMC Emergency Medicine
BMC Health Services Research
BMC Medical Ethics
BMC Molecular Biology
BMC Musculoskeletal Disorders
BMC Pregnancy and Childbirth
BMC Psychology
Behavior Research Methods, Instruments, & Computers
Best Practice & Research Clinical Gastroenterology
BioControl
BioIndustry Ethics
BioMedicine
Bioactive Materials

Bioanalytical Reviews
Biochimica et Biophysica Acta (BBA) - Gene Structure and Expression
Biochimica et Biophysica Acta (BBA) - Reviews on Cancer
Biogeochemistry
Biohydrogen
Biological Cybernetics
Biological Invasions
Biology & Philosophy
Biology and Fertility of Soils
Biology of Blood and Marrow Transplantation
Biomarkers in Toxicology
Biomedical Engineering Letters
Biomedical Engineering in Gastrointestinal Surgery
Biomedical Ethics for Engineers
Biomedical Information Technology
Biomedical Microdevices
Biomolecular Engineering
Biophysical Economics and Sustainability
Biophysics
Bioresources and Bioprocessing
Biotechnology for Biofuels
Biscuit Baking Technology
Blue Books of Neurology
Bluetooth Application Developer's Guide
Boletín de la Sociedad Española de Cerámica y Vidrio
Bonding Theory for Metals and Alloys
Borish's Clinical Refraction
Brain Mapping
Brain Mapping: The Systems
Brain Research Bulletin
Brain Research Reviews
Brain Stimulation
Brain Structure and Function
Brain and Language
Brittonia
Building Robots with LEGO Mindstorms NXT
Building Wireless Sensor Networks

Bulletin of Earthquake Engineering
Business Modeling and Data Mining
CABI Agriculture and Bioscience
CANON DSLR
CCF Transactions on Pervasive Computing and Interaction
CCTV Surveillance
CE Conformity Marking
Canadian Journal of Diabetes
Canadian Journal of Emergency Medicine
Cancer Gene Therapy
Canon EOS Digital Rebel XS/1000D
Carbon Dioxide Utilisation
Carbon Resources Conversion
Cardiovascular Intervention and Therapeutics
Cardiovascular Pathology
Cardiovascular Therapeutics
Case Studies in Thermal Engineering
Cases Journal
Cell Biology International
Cell Death & Differentiation
Cellular Response to Biomaterials
Chalcogenide Glasses
Chemoecology
Chinese Journal of Analytical Chemistry
Chinese Journal of Traumatology
Chinese Medicine
Cirugía Española (English Edition)
Cities
Cladistics
Clay-Polymer Nanocomposites
Climate Vulnerability
Clinical Assessment and Intervention for Autism Spectrum Disorders
Clinical Cardiac Pacing, Defibrillation, and Resynchronization Therapy
Clinical Dentistry Reviewed
Clinical Diabetes
Clinical Microbiology and Infection
Clinical Neurophysiology

Clinical Oral Investigations
Clinical Radiology
Clinical Reviews in Bone and Mineral Metabolism
Clinical Techniques in Equine Practice
Clinical and Translational Gastroenterology
Clinical and Translational Imaging
Clinical and Translational Oncology
Clinical and Translational Science
Coal Energy Systems
Cognitive Development
Cognitive Psychology
Cognitive and Behavioral Practice
Colloids and Interface Science Communications
Communications Physics
Communications in Nonlinear Science and Numerical Simulation
Complementary Therapies in Medicine
Complementary and Integrative Therapies for Cardiovascular Disease
Complete Casting Handbook
Composite Magnetoelectrics
Composites Part B: Engineering
Comprehensive Medicinal Chemistry III
Comprehensive Natural Products II
Comprehensive Organic Functional Group Transformations II
Comptes Rendus Chimie
Computational Statistics
Computer Methods in Applied Mechanics and Engineering
Computer-Aided Design
Computerized Medical Imaging and Graphics
Computers & Fluids
Computers & Graphics
Computers & Structures
Construction Hazardous Materials Compliance Guide
Contemporary Concepts of Condensed Matter Science
Contemporary Educational Psychology
Continental Shelf Research
Contraception
Contributions to Economic Analysis

Copper
Corporate Environmental Strategy
Cortex
Criminology
Crisis Negotiations
Crystallization
Current Atherosclerosis Reports
Current Behavioral Neuroscience Reports
Current Cardiology Reports
Current Climate Change Reports
Current Forestry Reports
Current HIV/AIDS Reports
Current Hematologic Malignancy Reports
Current Neurology and Neuroscience Reports
Current Oncology Reports
Current Opinion in Genetics & Development
Current Opinion in Neurobiology
Current Paediatrics
Current Problems in Diagnostic Radiology
Current Therapy of Trauma and Surgical Critical Care
Data Conversion Handbook
Data Science and Engineering
Deep Sea Research Part I: Oceanographic Research Papers
Demystifying Switching Power Supplies
Der Internist
Dermatology and Therapy
Designing Embedded Systems with PIC Microcontrollers
Development Genes and Evolution
Developmental Cognitive Neuroscience
Developments in Food Science
Developments in Surface Contamination and Cleaning: Applications of Cleaning Techniques
Diagnostic Immunohistochemistry
Dialogue Editing for Motion Pictures
Diet and Nutrition in Dementia and Cognitive Decline
Digital Compositing for Film and Video
Digital Signage
Dilute Nitride Semiconductors

Direct Alcohol Fuel Cells for Portable Applications
Directing and Producing for Television
Disasters and Public Health
Drinking Water Security for Engineers, Planners, and Managers
Drugs & Aging
Drugs, Addiction, and the Brain
Dynamics of Tethered Satellite Systems
EPMA Journal
ERCP
EURASIP Journal on Audio, Speech, and Music Processing
EURO Journal on Transportation and Logistics
Eating and Weight Disorders - Studies on Anorexia, Bulimia and Obesity
Ecological Indicators
Ecological Modelling
Economia Politica
Economic Modelling
Economics & Human Biology
Economía Informa
Ecotextiles
Ecotoxicology
Educational Assessment, Evaluation and Accountability
Effective Physical Security
Effective Security Management
Egyptian Journal of Ear, Nose, Throat and Allied Sciences
Egyptian Journal of Medical Human Genetics
Electoral Studies
Electrical Engineer's Reference Book
Electrical Engineering 101
Electrochemical Water and Wastewater Treatment
Electronic Enclosures, Housings and Packages
Elements of Orthopaedic Surgery
Embedded Media Processing
Embedded System Design on a Shoestring
Embedded Systems
Encyclopedia of Biomedical Engineering
Encyclopedia of Forensic and Legal Medicine
Encyclopedia of Gastroenterology

Encyclopedia of Human Behavior
Encyclopedia of Insects
Encyclopedia of Language & Linguistics
Encyclopedia of Social Measurement
Encyclopedia of Sustainable Technologies
Encyclopedia of the Eye
Endocrine
Endocrinología y Nutrición
Enfermería Clínica
Enfermería Clínica (English Edition)
Engineering
Entrepreneurs
Environmental Fluid Mechanics
Epilepsy & Behavior
Erwerbs-Obstbau
Esophagus
Essentials of Lean Six Sigma
Estuaries and Coasts
Ethanol
Eurasian Economic Review
European Archives of Psychiatry and Clinical Neuroscience
European Economic Review
European Journal of Cancer
European Journal of Clinical Nutrition
European Journal of Medical Genetics
European Journal of Medical Research
European Journal of Nutrition
European Journal of Paediatric Neurology
European Journal of Plastic Surgery
European Journal of Trauma & Dissociation
European Surgery
Evidence-Based Dentistry
Evidence-Based Educational Methods
Evolution: Education and Outreach
Exergy Analysis and Thermoeconomics of Buildings
Experimental Cell Research
Experimental Gerontology

Experimental Methods in the Physical Sciences
Experimental and Applied Acarology
Expert Systems with Applications
Exploring Engineering
Exposure and Health
Extremophiles
FEMS Microbiology Ecology
FISMA Compliance Handbook
Field Guide to Appropriate Technology
Filters and Filtration Handbook
Filtration Industry Analyst
Fine Chemicals Manufacture
Fire Protection Engineering in Building Design
Fire Pump Arrangements at Industrial Facilities
Fish Physiology
Fisioterapia
Fixed/Mobile Convergence and Beyond
FlatChem
Folia Microbiologica
Food Quality and Shelf Life
Food Science and Human Wellness
Forensic Science, Medicine and Pathology
Forensic Toxicology
Forest Policy and Economics
Foundations of Artificial Intelligence
Fractography in Failure Analysis of Polymers
Free Radical Biology and Medicine
From Neuroscience To Neurology
From Smart Grid to Internet of Energy
Functional Dyes
Functional Neurobiology of Aging
Functional Textiles for Improved Performance, Protection and Health
Fundamental Biomaterials: Ceramics
Fundamentals of Molecular Structural Biology
Fusion
Future of Utilities Utilities of the Future
GSTF Journal of Engineering Technology (JET)

Gene
General Philosophy of Science
Geoforum
Geopolymers
Geoscience Frontiers
Global Environmental Change Part B: Environmental Hazards
Gold Bulletin
Groundwater for Sustainable Development
Group Decision and Negotiation
HDTV and the Transition to Digital Broadcasting
HSS Journal ®
Habitat International
Handbook of Adhesives and Surface Preparation
Handbook of Advanced Ceramics
Handbook of Biomechatronics
Handbook of Biopolymers and Biodegradable Plastics
Handbook of Economic Growth
Handbook of Green Building Design and Construction
Handbook of Hydrothermal Technology
Handbook of Liquids-Assisted Laser Processing
Handbook of Models for Human Aging
Handbook of Nonwoven Filter Media
Handbook of Organizational Creativity
Handbook of Psychological Assessment
Handbook of Public Economics
Handbook of Solvents
Handbook on the Physics and Chemistry of Rare Earths
Handbook on the Toxicology of Metals
Hardware/Firmware Interface Design
Hazardous Chemicals Handbook
Health Research Policy and Systems
Health and Quality of Life Outcomes
Health and Technology
Heat Pipes
Hepatology
Hepatology Research
Hereditary Cancer in Clinical Practice

Heterogeneous Photocatalysis
High Frequency and Microwave Engineering
High Performance Textiles and their Applications
How to Cheat in Photoshop CS3
Human Cell
Human Genetics
Human Growth and Development
Human Immunology
Human Nature
Hybrid Polymer Composite Materials
Hydrogeology Journal
Hyperfine Interactions
IMF Economic Review
IMS Application Developer's Handbook
ISPRS Journal of Photogrammetry and Remote Sensing
ISSS Journal of Micro and Smart Systems
IST International Surface Technology
ITBM-RBM
IZA Journal of Labor & Development
Icarus
Image and Vision Computing
Immunotoxicology of Drugs and Chemicals: an Experimental and Clinical Approach
Improving Comfort in Clothing
In-Pack Processed Foods
Indian Journal of Orthopaedics
Indian Journal of Pediatrics
Indian Journal of Surgical Oncology
Industrial Crops and Products
Industrial Process Automation Systems
Infection
Information Economics and Policy
Information Fusion
Information Retrieval
Information and Computation
Infrared Physics & Technology
Infrastructure Complexity
Innovations in Fuel Economy and Sustainable Road Transport

Innovations in Systems and Software Engineering
Inorganica Chimica Acta
Instructional Science
Intelligent Industrial Systems
Interest Groups & Advocacy
Internal Combustion Engines: Performance, Fuel Economy and Emissions
International Advances in Economic Research
International Encyclopedia of Public Health
International Immunopharmacology
International Journal for Educational and Vocational Guidance
International Journal of Accounting Information Systems
International Journal of Advanced Structural Engineering
International Journal of Applied Positive Psychology
International Journal of Child Care and Education Policy
International Journal of Coal Science & Technology
International Journal of Disaster Risk Science
International Journal of Fatigue
International Journal of Food Microbiology
International Journal of Geo-Engineering
International Journal of Industrial Ergonomics
International Journal of Information Management
International Journal of Machine Tools and Manufacture
International Journal of Nursing Studies
International Journal of Osteopathic Medicine
International Journal of Parallel Programming
International Journal of Pediatric Endocrinology
International Journal of Politics, Culture, and Society
International Journal of Retina and Vitreous
International Journal of Rock Mechanics and Mining Sciences
International Journal of Trauma Nursing
International Journal on Software Tools for Technology Transfer
International Orthopaedics
International Review of Research in Mental Retardation
Introduction to Digital Systems
Invertebrate Neuroscience
Investigational New Drugs
Ionic Liquids in Separation Technology

Iran Journal of Computer Science
Irrigation Science
Israel Journal of Health Policy Research
Izvestiya, Atmospheric and Oceanic Physics
JACC: Cardiovascular Imaging
JACC: Cardiovascular Interventions
JETP Letters
Jornal de Pediatria
Journal für Verbraucherschutz und Lebensmittelsicherheit
Journal of Abnormal Child Psychology
Journal of Advanced Research
Journal of Agricultural and Environmental Ethics
Journal of Air Transport Management
Journal of Anesthesia History
Journal of Animal Science and Biotechnology
Journal of Animal Science and Technology
Journal of Behavioral Medicine
Journal of Big Data
Journal of Biochemical and Biophysical Methods
Journal of Biological Engineering
Journal of Biomedical Research
Journal of Bioscience and Bioengineering
Journal of Biosciences
Journal of Business Research
Journal of Business Venturing
Journal of Cardiology
Journal of Cardiothoracic Surgery
Journal of Catalysis
Journal of Cataract & Refractive Surgery
Journal of Cell Communication and Signaling
Journal of Cereal Science
Journal of Chemical Neuroanatomy
Journal of China University of Geosciences
Journal of Circadian Rhythms
Journal of Clinical Densitometry
Journal of Communications and Information Networks
Journal of Comparative Economics

Journal of Compassionate Health Care
Journal of Computer-Aided Molecular Design
Journal of Constructional Steel Research
Journal of Consumer Psychology
Journal of Contemporary Psychotherapy
Journal of Contextual Behavioral Science
Journal of Crohn's and Colitis
Journal of Crop Science and Biotechnology
Journal of Cultural Heritage
Journal of Cystic Fibrosis
Journal of Diabetes and its Complications
Journal of Ecology and Environment
Journal of Economic Dynamics and Control
Journal of Economics and Finance
Journal of Economics, Race, and Policy
Journal of Engineering Mathematics
Journal of Environmental Radioactivity
Journal of Ethnopharmacology
Journal of Experimental Child Psychology
Journal of Experimental Marine Biology and Ecology
Journal of Exposure Science & Environmental Epidemiology
Journal of Failure Analysis and Prevention
Journal of Fluency Disorders
Journal of Foot and Ankle Research
Journal of Friction and Wear
Journal of Global Entrepreneurship Research
Journal of Happiness Studies
Journal of Hospitality, Leisure, Sport & Tourism Education
Journal of Huazhong University of Science and Technology [Medical Sciences]
Journal of Industrial Microbiology and Biotechnology
Journal of Infection and Chemotherapy
Journal of Inflammation
Journal of Information Security and Applications
Journal of Integrative Agriculture
Journal of Investigative Dermatology
Journal of Light Metals
Journal of Luminescence

Journal of Management
Journal of Manipulative and Physiological Therapeutics
Journal of Maritime Archaeology
Journal of Materials Science: Materials in Medicine
Journal of Mathematics Teacher Education
Journal of Mathematics in Industry
Journal of Medical Systems
Journal of Men's Health
Journal of Microscopy and Ultrastructure
Journal of Midwifery & Women's Health
Journal of Modern Transportation
Journal of Molecular Neuroscience
Journal of Multinational Financial Management
Journal of Neonatal Nursing
Journal of Network and Computer Applications
Journal of Network and Systems Management
Journal of Neural Transmission
Journal of Neuroinflammation
Journal of Nuclear Cardiology
Journal of Ocean Engineering and Science
Journal of Ornithology
Journal of Orthopaedic Nursing
Journal of Orthopaedics and Traumatology
Journal of Otolaryngology - Head & Neck Surgery
Journal of Ovarian Research
Journal of Parallel and Distributed Computing
Journal of Pediatric and Adolescent Gynecology
Journal of Petroleum Exploration and Production
Journal of Plant Biochemistry and Biotechnology
Journal of Plant Growth Regulation
Journal of Plant Pathology
Journal of Plant Research
Journal of Plastic, Reconstructive & Aesthetic Surgery
Journal of Police and Criminal Psychology
Journal of Product Innovation Management
Journal of Prosthodontic Research
Journal of Proteomics

Journal of Psychopathology and Behavioral Assessment
Journal of Psychosocial Rehabilitation and Mental Health
Journal of Psychosomatic Research
Journal of Religious Education
Journal of Retailing
Journal of Science and Medicine in Sport
Journal of Science in Sport and Exercise
Journal of Sport and Health Science
Journal of Statistical Planning and Inference
Journal of Surgical Research
Journal of Sustainable Mining
Journal of Taibah University for Science
Journal of Tissue Viability
Journal of Translational Medicine
Journal of Urban Health
Journal of Veterinary Behavior
Journal of Virological Methods
Journal of Visual Communication and Image Representation
Journal of Volcanology and Seismology
Journal of Web Semantics
Journal of Zhejiang University-SCIENCE B
Journal of the American Oil Chemists' Society
Journal of the Brazilian Computer Society
Journal of the Chemical Society, Dalton Transactions
Journal of the Knowledge Economy
Journal of the National Medical Association
Journal on Data Semantics
Keeping Religious Institutions Secure
Killer Camera Rigs That You Can Build
Kinetics and Catalysis
Kirk and Bistner's Handbook of Veterinary Procedures and Emergency Treatment
Korean Social Science Journal
La Rivista del Nuovo Cimento
La radiologia medica
Laboratory Animal Medicine
Labour Economics
Landscape Ecology

Langford's Basic Photography
Le Praticien en Anesthésie Réanimation
Lead-Acid Batteries: Science and Technology
Learning and Memory: A Comprehensive Reference
Legal Aspects of General Dental Practice
Library Collections, Acquisitions, and Technical Services
Lighting Engineering
Lightweight Design worldwide
Lipids
Lipids in Health and Disease
Logistics Operations and Management
Low Back Pain Handbook
MRS Internet Journal of Nitride Semiconductor Research
Managing Wine Quality
Managing and Securing a Cisco SWAN
Manufacture
Marine Geophysical Researches
Marine Life Science & Technology
Marine Systems & Ocean Technology
Material Selection for Thermoplastic Parts
Materials in Sports Equipment
Mathematics Education Research Journal
Mathematics and Computers in Simulation
Mathematics in Science and Engineering
Maturitas
McDonald and Avery's Dentistry for the Child and Adolescent
Measuring Ocean Currents
Meccanica
Mechatronics for Safety, Security and Dependability in a New Era
Medical Oncology
Medical Robotics
Medical and Healthcare Textiles
Medicina Intensiva (English Edition)
Medizinische Klinik - Intensivmedizin und Notfallmedizin
Membrane Technology
Membranes for Clean and Renewable Power Applications
Metal Finishing

Metal Powder Report
Metals and Materials
Methods in Microbiology
Micro-Manufacturing Engineering and Technology
Microbial Cell Factories
Microbial Electrochemical Technology
Microbial Forensics
Microbiological Research
Microwave Wireless Communications
Mineral Exploration
Mixing a Musical
Modeling of Chemical Kinetics and Reactor Design
Modern Rheumatology
Molecular Autism
Molecular Breeding
Molecular Medical Microbiology
Molecular Metabolism
Molecular Oncology
Molecular Pain
Molecular Phylogenetics and Evolution
Molecular Therapy - Nucleic Acids
Molecular and Cellular Pediatrics
Monatshefte für Chemie - Chemical Monthly
Motor Vehicle Collisions
Multi-Camera Networks
Multidisciplinary Respiratory Medicine
Multilayer Flexible Packaging
Multimedia Tools and Applications
Multiprocessor Systems-on-Chips
Multisensory Perception
Mutation Research/Genetic Toxicology and Environmental Mutagenesis
Mycological Research
Mycopathologia
Myelin Biology and Disorders
NDT & E International
Nano Convergence
NanoEthics

Nanoarchitectonics in Biomedicine
Nanolayer Research
Nanostructured Metals and Alloys
Nanostructures for Drug Delivery
Nanotechnology Applications for Tissue Engineering
Nanotechnology Safety
Natural Computing
Natural Language & Linguistic Theory
Nature Astronomy
Nature Chemical Biology
Nature Clinical Practice Neurology
Nature Machine Intelligence
Nature Medicine
Nature Metabolism
Nature Methods
Nature Neuroscience
Nature Reviews Cardiology
Nature Reviews Neurology
Navigating the Maze
Nerves and Nerve Injuries
Network Modeling Analysis in Health Informatics and Bioinformatics
Networking Explained
Networks and Spatial Economics
Networks on Chips
Neurochemical Research
Neurochirurgie
Neurofeedback and Neuromodulation Techniques and Applications
Neurogenetics
Neurology and Clinical Neuroscience
Neurología Argentina
Neuropharmacology
Neuroscience
Neuroscience and Behavioral Physiology
Neurosensory Disorders in Mild Traumatic Brain Injury
Neurosurgical Review
New Trends in Coal Conversion
Newnes Guide to Radio and Communications Technology

Nikon D60
Non-Covalent Interactions in Quantum Chemistry and Physics
Non-Thermal Plasma Technology for Polymeric Materials
Nonclinical Development of Novel Biologics, Biosimilars, Vaccines and Specialty Biologics
Nonlinear Analysis: Theory, Methods & Applications
Notes on Chronic Otorrhœa
Nutraceuticals
Obesity Surgery
Obstetrics, Gynaecology & Reproductive Medicine
Ocean Engineering
Ocular Therapeutics
One Health Outlook
Open Source Software
Operative Techniques in Neurosurgery
Optical Fiber Technology
Optical and Quantum Electronics
Optimization Letters
Optometry - Journal of the American Optometric Association
Oral Oncology Supplement
Oral Science International
Organic and Medicinal Chemistry Letters
Orthopaedic Physical Therapy Secrets
Otologic Surgery
PAIN
PEM Fuel Cell Modeling and Simulation Using Matlab
Paediatrics and Child Health
Paleontological Journal
Palgrave Communications
Parasitoid Viruses
Passive Optical Networks
Pathology
Pediatric Cardiology
Pediatric Critical Care
Pediatric Neurology
Pediatric Rheumatology
Perception & Psychophysics
Perfect Passwords

Performance Evaluation
Perioperative Nursing Clinics
Permeability Properties of Plastics and Elastomers
Personal Safety and Security Playbook
Perspectives in Medicine
Perspectives in Plant Ecology, Evolution and Systematics
Perspectives on Behavior Science
Pervasive and Mobile Computing
Pharmaceuticals and Personal Care Products: Waste Management and Treatment Technology
PharmacoEconomics - Open
Pharmacology & Therapeutics
Philosophy of Technology and Engineering Sciences
Philosophy, Ethics, and Humanities in Medicine
Photonic Network Communications
Photoshop CS3 Essential Skills
Photovoltaics Bulletin
Physical Medicine and Rehabilitation Clinics of North America
Physical Techniques in the Study of Art, Archaeology and Cultural Heritage
Physical and Engineering Sciences in Medicine
Physical and Logical Security Convergence
Physics in Perspective
Physics of Particles and Nuclei
Pneumonia
Polyurethane Polymers
Population Research and Policy Review
Porous Silicon for Biomedical Applications
Power Plant Instrumentation and Control Handbook
Practical Electrical Equipment and Installations in Hazardous Areas
Practical Fiber Optics
Practical Guide to Clinical Computing Systems
Practical Power System and Protective Relays Commissioning
Practical Reservoir Engineering and Characterization
Pramana
Preventive Veterinary Medicine
Primary Care Diabetes
Primer on the Autonomic Nervous System
Principles and Applications of RF/Microwave in Healthcare and Biosensing

Principles and Practice of Clinical Research
Principles of Addiction
Principles of Clinical Pharmacology
Principles of Measurement and Transduction of Biomedical Variables
Procedia - Social and Behavioral Sciences
Procedia IUTAM
Proceedings of the National Academy of Sciences, India Section B: Biological Sciences
Product Development
Progress in Heterocyclic Chemistry
Progress in Natural Science
Project Finance in Theory and Practice
Properties and Performance of Natural-Fibre Composites
Propulsion and Power Research
Psychological Research
Psychology in the Physical and Manual Therapies
Psychopathologie transculturelle
Psychosocial Conceptual Practice Models in Occupational Therapy
Publishing Research Quarterly
Pulmonary Pharmacology & Therapeutics
Quality & Quantity
Quality Control and Evaluation of Herbal Drugs
Quaternary Research
RF Engineering for Wireless Networks
Rabies
Radiation Physics and Chemistry
Radio Frequency Transistors
Radioactivity
Radiochemistry
Railway Engineering Science
Rangeland Ecology & Management
Reaction Chemistry & Engineering
Real-Time UML Workshop for Embedded Systems
Recent Advances in Thermo-Chemical Conversion of Biomass
Recent Developments in Applied Electrostatics
Reconfigurable Computing
Redox Biology
Reflow Soldering Processes

Regional Studies in Marine Science
Regulatory Toxicology and Pharmacology
Reproductive Health Matters
Res Publica
Research Proposals
Research in Developmental Disabilities
Research in Economics
Research in Higher Education
Research in Microbiology
Research in Organizational Behavior
Research in Transportation Business & Management
Resonance
Resource-Efficient Technologies
Respiratory Research
Results in Physics
Review of Evolutionary Political Economy
Review of Quantitative Finance and Accounting
Reviews in Fish Biology and Fisheries
Reviews of Modern Plasma Physics
Revista Española de Cardiología Suplementos
Revista Española de Medicina Nuclear (English Edition)
Revista de Gastroenterología de México (English Edition)
Revista de Psicodidáctica (English ed.)
Revue Européenne de Psychologie Appliquée/European Review of Applied Psychology
Revue Francophone d'Orthoptie
Revue Neurologique
Rice
Rock Mechanics and Rock Engineering
Rules of Thumb for Maintenance and Reliability Engineers
Russian Agricultural Sciences
Russian Engineering Research
Russian Journal of Coordination Chemistry
SAE and the Evolved Packet Core
SN Computer Science
Sadhana
Safety and Security Review for the Process Industries
Safety in Extreme Environments

Scandinavian Journal of Trauma, Resuscitation and Emergency Medicine
Schizophrenia Research
School Mental Health
Scientia Iranica
Scientific Phone Apps and Mobile Devices
Security Informatics
Security and Resilience in Intelligent Data-Centric Systems and Communication Networks
Semiconductor Gas Sensors
Seminars in Pediatric Infectious Diseases
Sensors for Health Monitoring
Sexuality Research and Social Policy
Shock Waves
Silicon Carbide Biotechnology
Silicon-On-Insulator (SOI) Technology
Simulation Modelling Practice and Theory
Singapore Dental Journal
Sintering of Advanced Materials
Sleep Medicine
Sleep and Breathing
Small Wind
Small-scale Forestry
Smart Textiles and their Applications
Smart Things
Smart Wheelchairs and Brain-Computer Interfaces
Social Anxiety
Social Choice and Welfare
Social Indicators Research
Sodium Sulfate
Software Engineering for Embedded Systems
Soil Biology and Biochemistry
Solar System Research
Solid Waste Recycling and Processing
Solvent Extraction
Somnologie
Sony A300/A350
South African Journal of Botany
Speedlights & Speedlites

Sport Management Review
Sport, Recreation and Tourism Event Management
Sport-Orthopädie - Sport-Traumatologie - Sports Orthopaedics and Traumatology
Statistics and Computing
Statistics in Biosciences
Stem Cell Research & Therapy
Storyboards
Structural Health Monitoring of Civil Infrastructure Systems
Studies in East European Thought
Studies in History and Philosophy of Science Part A
Studies in Multidisciplinarity
Studies in Philosophy and Education
Superconductors in the Power Grid
Supportive Care in Cancer
Surface Chemistry of Nanobiomaterials
Surface Science Reports
Surfaces and Interfaces
Surgical Endoscopy And Other Interventional Techniques
Surgical and Experimental Pathology
Sustainable Cities and Society
Sustainable Design and Build
Sustainable Water and Wastewater Processing
System
System Requirements Analysis
Systems Microbiology and Biomanufacturing
TOP
Targeted Oncology
Tea in Health and Disease Prevention
Technology in Society
Technology, Humans, and Society
Technovation
Tetraplegia and Paraplegia
Textiles in Automotive Engineering
Thalassas: An International Journal of Marine Sciences
The American Journal of Geriatric Pharmacotherapy
The American Journal of Medicine
The Arts in Psychotherapy

The Assessment and Treatment of Addiction
The Best Damn Cisco Internetworking Book Period
The Biology of Human Longevity
The Case Manager
The Cloud Security Ecosystem
The Concise Focal Encyclopedia of Photography
The Effective Security Officer's Training Manual
The Egyptian Journal of Neurology, Psychiatry and Neurosurgery
The European Physical Journal C
The European Physical Journal D
The Geneva Papers on Risk and Insurance - Issues and Practice
The Geochemical Society Special Publications
The HLA FactsBook
The IBOC Handbook
The Joint Commission Journal on Quality and Safety
The Journal of Arthroplasty
The Journal of Behavioral Health Services & Research
The Journal of Foot and Ankle Surgery
The Journal of Hand Surgery: British & European Volume
The Journal of Heart and Lung Transplantation
The Journal of Mathematical Neuroscience
The Journal of Membrane Biology
The Journal of Prevention of Alzheimer's Disease
The Journal of Primary Prevention
The Journal of Real Estate Finance and Economics
The Journal of Steroid Biochemistry and Molecular Biology
The Journal of VLSI Signal Processing Systems for Signal, Image, and Video Technology
The Journal of Value Inquiry
The Journal of the American Dental Association
The Journal of the Astronautical Sciences
The Laboratory Fish
The MPEG Handbook
The Mobile Connection
The Multibody Systems Approach to Vehicle Dynamics
The Performance of Photovoltaic (PV) Systems
The Principles of Experimental Research
The Psychology of Serial Killer Investigations

The ROV Manual
The Real Life Guide to Accounting Research
The SGTE Casebook
The Science of Fitness
The Urban Review
Theoretical Computer Science
Theoretical and Applied Climatology
Theory and Society
Therapy in Sleep Medicine
Thermochimica Acta
Thermodynamics
Thermoforming of Single and Multilayer Laminates
Thermoplastics and Thermoplastic Composites
Thermosets and Composites
Toxicology Research
Transactions of the Indian National Academy of Engineering
Transfusion Medicine and Hemostasis
Translational Respiratory Medicine
Transplant International
Transport Properties of Concrete
Transportation Research Part B: Methodological
Transportation Research Part E: Logistics and Transportation Review
Treatise on Estuarine and Coastal Science
Treatment of the Postmenopausal Woman
Trees
Trends in Food Science & Technology
Trends in Immunology
Trends in Neuroscience and Education
Tribology Series
Tribology and Interface Engineering Series
Türk Fizyoterapi ve Rehabilitasyon Dergisi/Turkish Journal of Physiotherapy and Rehabilitation
URBAN DESIGN International
Underground Infrastructures
Understanding Automotive Electronics
Urologic Surgical Pathology
User Experience in the Age of Sustainability
User Modeling and User-Adapted Interaction

Vehicle Thermal Management Systems Conference and Exhibition (VTMS10)
Veterinary Immunology and Immunopathology
Veterinary Parasitology: Regional Studies and Reports
Vibrational Spectroscopy
Video Journal and Encyclopedia of GI Endoscopy
Video Shooter
Virtual Reality
Vision Research
Vital
WISC-V Assessment and Interpretation
Waste Engine Oils
Waste Management
Water Resources Management
Water, Air, & Soil Pollution: Focus
Welded Design
Well Logging and Formation Evaluation
Wide Bandgap Semiconductor Power Devices
Wills' Mineral Processing Technology
Wine Science
World Development
World Development Perspectives
World Journal of Pediatrics
Zeitschrift für Vergleichende Politikwissenschaft
Zen and the Art of Information Security
